# Does interference between self and other perspectives in theory of mind tasks reflect a common underlying process? Evidence from individual differences in theory of mind and inhibitory control

**DOI:** 10.3758/s13423-019-01656-z

**Published:** 2019-08-19

**Authors:** Adam W. Qureshi, Rebecca L. Monk, Dana Samson, Ian A. Apperly

**Affiliations:** 1 Edge Hill University, Ormskirk, L39 4PY UK; 2 Université Catholique de Louvain, L0.01.03, 1348 Louvain-la-Neuve, Belgium; 3 University of Birmingham, Edgbaston, B15 2TT UK

**Keywords:** Theory of mind, Executive function, Self–other interference, Path analysis

## Abstract

Theory of mind (ToM), the ability to understand that other agents have different beliefs, desires, and knowledge than oneself, has been extensively researched. Theory of mind tasks involve participants dealing with interference between their self-perspective and another agent’s perspective, and this interference has been related to executive function, particularly to inhibitory control. This study assessed whether there are individual differences in self–other interference, and whether these effects are due to individual differences in executive function. A total of 142 participants completed two ToM (the director task and a Level 1 visual perspective-taking task), which both involve self–other interference, and a battery of inhibitory control tasks. The relationships between the tasks were examined using path analysis. Results showed that the self–other interference effects of the two ToM tasks were dissociable, with individual differences in performance on the ToM tasks being unrelated and performance in each predicted by different inhibitory control tasks. We suggest that self–other differences are part of the nature of ToM tasks, but self–other interference is not a unitary construct. Instead, self–other differences result in interference effects in various ways and at different stages of processing, and these effects may not be a major limiting step for adults’ performance on typical ToM tasks. Further work is needed to assess other factors that may limit adults’ ToM performance and hence explain individual differences in social ability.

**Electronic supplementary material:**

The online version of this article (10.3758/s13423-019-01656-z) contains supplementary material, which is available to authorized users.

Theory of mind (ToM) has been defined as the ability to understand that other agents have different beliefs, desires, and knowledge than oneself (Premack & Woodruff, 1978). While neurotypical adults clearly understand the principle that other people may have a different perspective than themselves (perhaps unlike children; e.g., Perner & Ruffman, 2005; though see Onishi & Bailargeon, 2005), they nonetheless are prone to systematic difficulties arising from conflict between their own perspective (self-perspective) and another’s. Most notably, adult participants show “egocentrism” across a wide range of tasks, whereby their judgment of what someone else sees, thinks, or wants is slower or more error prone when this differs from what participants themselves see, think, or want (Royzman, Cassidy, & Baron, 2003). Understanding the nature and origin of such interference effects is critical for theories about the functional bases of ToM, and understanding variability in these effects between individuals might help to explain why some people are more socially able than others (e.g., Apperly, 2012). Despite the recent proliferation of tasks available for studying ToM in adults, little is yet known about individual differences in ToM performance. Therefore, this study addresses two fundamental questions: Are there systematic individual differences in self–other interference, and are these effects due to individual differences in executive functions?

Studies of ToM in adults have focused on both typical and atypical participants. Specifically, research has examined healthy young adults (e.g., Bradford, Jentzsch, & Gomez, 2015; Qureshi & Monk, 2018) and older adults (Bernstein, Thornton, & Sommerville, 2011), patients with brain injury (e.g., Apperly, Samson, & Humphreys, 2005; Stone, Baron-Cohen, & Knight, 1998), those with autism spectrum disorder (e.g., Baron-Cohen, Jolliffe, Mortimore, & Robertson, 1997), psychopathy (e.g., Lockwood, Bird, Bridge, & Viding, 2013), and dementia (e.g., Le Bouc et al., 2012). Research in this area has also utilized various methodologies, including dual tasking (e.g., Bull, Phillips, & Conway, 2008; Qureshi, Apperly, & Samson, 2010), brain stimulation (e.g., Kalbe et al., 2010), neuroimaging (e.g., Reiniers, Völlm, Elliott, & Corcoran, 2014), and individual differences (Ryskin, Benjamin, Tullis, & Brown-Schmidt, 2015). Nevertheless, a unifying feature of the tasks is that they generate a difference between the perspective of the participant (self-perspective) and that of the target (other-perspective), with the resulting interference between the self and other perspectives requiring resolution to judge what the other thinks, sees, or feels. This gives rise to the widely reported phenomenon of “egocentric bias” towards the participant’s own perspective, which is almost universally observed in studies of ToM (Royzman et al., 2003; Wellman, 2014). However, it is currently unclear whether self–other interference results from the same underlying functional process on different tasks. While everyday experience lends credibility to the hypothesis that some individuals are consistently more egocentric than others, few studies have examined whether this is the case.

One leading hypothesis is that interference between self and other perspectives in ToM tasks forms part of a broader domain of phenomena in which representations relating to self and other must be controlled (e.g., Cook, 2014). For example, observing another’s action generates a tendency for “automatic imitation” of the action by oneself, which must be controlled if it is not the action required by the task or ongoing activity (Brass, Bekkering, Wohlschläger, & Prinz, 2000). Santiesteban et al. (2012) found that training participants to inhibit automatic imitation improved performance on a test that required participants to use their ToM (the director task; Keysar, Lin, & Barr, 2003), whereas training generic inhibition did not, suggesting that inhibition of automatic imitation and ToM rely on control over representations related to self and other, and these control processes are not the same as those involved in conventional “inhibition” tasks. Moreover, self–other control appears to depend on regions of the medial prefrontal cortex and temporoparietal cortex that are distinct from more lateral prefrontal brain regions frequently implicated in analogous nonsocial executive control (Bardi, Six, & Brass, 2017; Brass, Ruby, & Spengler, 2009; Brass, Derrfuss, & von Cramon, 2005; Wagner, Maril, Bjork, & Schacter, 2001). Such findings suggest that the same self–other control process may underlie both imitation inhibition and perspective taking, and that self–other control processes are in an important sense “domain-specific” and distinct from domain-general executive functions (as generic inhibition training did not improve ToM performance; though, see a recent meta-analysis by Darda & Ramsey, 2019). This clearly leads to the prediction that there will be reliable individual differences in self–other control across different ToM tasks, and that these should not be related to individual differences in generic executive function.

However, substantial evidence suggests that domain-general executive function is involved in ToM, and in particular in self–other control. Research on both children (Carlson & Moses, 2001; Carlson, Moses, & Breton, 2002; Carlson, Moses, & Claxton, 2004) and adults (Bradford et al., 2015; German & Hehman, 2006) has shown a relationship between executive function and ToM, and this has been suggested to explain deficits and impairments in ToM performance (Bull et al., 2008; McKinnon & Moscovitch, 2007). Although there is some evidence of a role for working memory (e.g., Qureshi & Monk, 2018; Ryskin et al., 2015), inhibitory control has been frequently linked with enabling the participant to control interference between self and other perspectives in order to select the one required by the task. For example, Qureshi et al. (2010) found that a secondary task taxing inhibitory control disproportionately impaired perspective taking on trials involving self–other conflict compared with trials without conflict (see also Todd, Cameron, & Simpson, 2017). Moreover, there is converging evidence from research using EEG, fMRI, neurospsychological studies of patients with brain injury, as well as TMS, that the inferior frontal gyrus—a brain region frequently implicated in generic inhibitory control—is involved in managing self–other conflict when it arises during visual perspective taking (e.g., McCleery, Surtees, Graham, Richards, & Apperly, 2011; Ramsey et al., 2013) and reasoning about false beliefs (e.g., Hartwright, Hansen, & Apperly, 2016; Samson, Apperly, Kathirgamanathan, & Humphreys, 2005; Samson et al., 2015). This evidence leads to the prediction that individual differences in self–other control on ToM tasks could be related to individual differences in generic executive control (in particular, in inhibitory control). This evidence would also be consistent with reliable correlations in self–other control across different ToM tasks, but such relationships should be mediated by individual differences in domain-general executive control, rather than by any specific process for self–other control. Importantly, this way of thinking about ToM tasks also allows for the possibility that although self–other control is typically assumed to be a coherent process common to all ToM tasks, egocentrism and other self–other interference effects are instead a “family” of phenomena that can arise in different ways across different tasks or situations. On such an account self–other interference effects may not correlate across different ToM tasks, but would correlate with executive function tasks. The pattern of those correlations would then depend upon when and how self–other interference arises during the ToM task, and the particular executive demands that arose as a result.

In the largest individual differences study of ToM to date, the current study therefore examines the relationship between performance on inhibitory control tasks and two ToM tasks that both involve dealing with interference between self and other perspectives. The two ToM tasks used were a Level 1 visual perspective-taking (L1 VPT) task (Samson, Apperly, Braithwaite, & Andrews, 2010) and the director task (based on the experiment of Keysar et al., 2003).

The L1 VPT task requires participants to judge how many dots appear on the walls of a room, either from their own perspective or from the perspective of an avatar in the room. This involves control processes relating to the ability to regulate the choice of responses (e.g., perspective selection) as well as more automatic processes that are associated with the calculation of an “other” perspective (see Qureshi et al., 2010). In terms of individual differences, this task has recently been used to create separate measures of “conflict” (relating to the interference between self and other perspectives) and “focus” (relative ease of judgements relating to self vs. other; Bukowski & Samson, 2017). These measures allow the separation of two components that may contribute to successful perspective taking, but which are confounded in standard measures of speed or accuracy of judging the perspectives of others.

The director task involves the participant following the instructions of a director who has a spatially opposite perspective to their own, and moving objects that are mutually visible (target objects) while avoiding objects visible only to the participant (competitor objects; Apperly et al., 2010). This task therefore also requires participants to resolve interference between self and other perspectives, though errors appear to arise not from difficulty with taking the director’s perspective, but with integrating this perspective with the director’s message in order to constrain reference (Barr, 2008).

Path analyses were used to model the relationship between four inhibitory control tasks and measures of ToM tasks. Given the near ubiquity of egocentric effects and other self–other interference effects, it is plausible that these effects reflect a common underlying process, with shared variance such that someone who shows (say) high self–other interference on one task will also show high interference on another. Prediction 1: This observation predicts a significant path between measures of self–other interference in the different ToM tasks—an important prediction that has received little empirical attention to date (see Ryskin et al., 2015, for an exception in the context of perspective taking during communication). Prediction 2: If inhibitory control is required for dealing with self–other perspective interference and egocentrism (failures to inhibit self-perspective), paths would be expected between inhibitory control and ToM. Prediction 3: If Predictions 1 and 2 are both supported, then the domain-general account of self–other control predicts that the relationships between self–other interference on different ToM tasks will be fully mediated by inhibitory control, whereas the domain-specific account of self–other control predicts that it will not be mediated. Prediction 4: If only Prediction 2 is supported, and if self–other interference arises in different ways for different ToM tasks, then self–other interference on different ToM tasks might correlate with different inhibition tasks.

## Method

### Participants

One hundred and fifty-four university student participants took part in the study for course credits or cash payment. Participant ages ranged from 18 to 44 years (*M* = 21.8 years, *SD* = 4.4 years), with 31 males and seven who were left-handed (two of whom used their right hand normally). Ethical approval was gained from the departmental ethics committee.

### Materials

All the experiments were designed and presented on a 15-inch Samsung SyncMaster 793s monitor connected to a 3.00 GHz Pentium-based desktop PC using E-Prime 1.1 (Schneider, Eschmann, & Zuccolotto, 2002), apart from the visual perspective-taking task which was presented using DMDX (Forster & Forster, 2003). A standard 102 keyboard was used for responses.

### Inhibitory control tasks

Based on Friedman and Miyake (2004), we chose tasks that required inhibition at different stages of processing, including resistance to distracting stimuli, resistance to distracting information held in working memory, and resistance to selecting a prepotent but incorrect response. Further details on the choice of tasks (A1), task methodology (A2), and all individual task results (A3) are shown in Appendix A, where two further inhibitory control tasks are described, a Simon task and a cued-recall task.^1^ These were not included in the final analyses due to having no relationship with any of the other tasks in the model and low reliability, respectively.

#### Shape matching

Participants were shown a green target shape and a white shape (see DeSchepper & Treisman, 1996). On 50% of trials there was a red distracter shape placed over the target. Participants were asked to decide if the target matched the white shape. The dependent variable for the path analysis model was the difference in response times (to correct trials) between the two distracter-present conditions and the two distracter-absent conditions ((distracter present match + distracter present no-match)/2 − (distracter absent match + distracter absent no-match)/2)).

#### Stop signal

Participants were presented with stimuli (“O” or “X”) and instructed to respond by pressing the same key on the keyboard (go trials: 75% of all trials), apart from stop trials (25%)—trials in which a tone was heard at a set delay (stop-signal delay; SSD) after presentation of the stimulus. The SSD followed a dynamic tracking procedure starting at 250 ms, and increased by 50 ms on a correct (non-)response, and decreased by 50 ms on an incorrect response (to stop trials). Stop-signal reaction time (SSRT) was calculated as mean Go RT − mean Stop RT.

#### Go/no-go (letter based)

Participants were shown serially presented letters on-screen that they were required to respond to by pressing the space bar (go trials; Wager et al., 2005). This was the requirement for all letters except for the letter *K* (no-go trial; 13.85% of all trials). The dependent variable was the false-alarm rate (FAR) for no-go trials.

#### Go/no-go (image based)

This task was based on the study of Schmitt, Münte, and Kutas (2000) and required a two-step process to respond correctly: The initial step was based on semantic information (image of either a bird or a mammal) and the go/no-go step on phonological or orthographical information (go trial = initial letter was a consonant; no-go trial = initial letter was a vowel; van Turennout, Hagoort, & Brown, 1997). The measure taken was the FAR to no-go trials.

### ToM tasks

Visual perspective taking is widely regarded as an important component of children’s developing ToM (e.g., Wellman, 2014) and of the mature ToM abilities of adults (e.g., Apperly, 2010). We selected two tasks that have been widely used in laboratory investigations of ToM in adults, and for which there were task analyses of component processes to which inhibition might contribute: the L1 VPT task requires calculation of another person’s visual perspective; the director task requires calculation of another person’s perspective, plus the use of this information to interpret their instructions. In the Discussion we evaluate the implications for the present work of alternative interpretations of these tasks.

#### L1 VPT task

The visual perspective-taking task used the stimuli and procedure of Experiment 1 in Samson et al. (2010). The stimuli consisted of a picture showing a lateral view into a room with the left, back, and right walls visible and with red dots displayed on one or two walls (stimuli were created using the 3-D animation program Poser 6©, Curious Lab). A centrally positioned human avatar faced either the left or right wall (see Table 2). On 50% of trials the avatar’s position meant that he or she saw the same dots as the participants (“consistent” condition). On 50% of trials, the avatar’s position meant that he or she could not see some of the dots that were visible to the participants (“inconsistent” condition). Indices of conflict (inconsistent–consistent perspectives) and focus (self-perspective–other perspective) were calculated as per Bukowski and Samson (2017). Inverse efficiency scores (IES; response time / 1 − error rate) were calculated for these conditions.^2^ A higher value in the conflict index indicated greater difficulty in handling conflicting perspectives, while positive values in the focus index indicated better performance in taking the other person’s perspective than the self- perspective (more altercentric rather than egocentric).

#### Director task

In a 4 × 4 experimental grid, a critical instruction was given by the director that could refer to a target object (mutually visible to the participant and instructor), or to a competitor object (located in one of the covered slots, and so visible only to the participant). In order to choose the target object, the participant needed to consider the instructor’s perspective. The remaining objects in the grid were unrelated. In the equivalent control grids, the competitor object was replaced by another unrelated object.

The measure taken was the number of errors made to ambiguous trials (where the item in the instruction could refer to a competitor and a target object, such as “mouse,” referring to either a computer mouse or a small mammal, for instance) and relational trials (where the best fitting object to the instruction was not mutually visible—for example, referring to a “small ball,” where the smallest ball was a golf ball only visible to the participant, but the smallest mutually visible ball was a tennis ball).

#### Task order

The order of the tasks was fixed so that participants were exposed to identical stimulus contexts (stimuli and order; see Carlson & Moses, 2001; Carlson et al., 2004; Friedman & Miyake, 2004; Miyake et al., 2000; Miyake, Friedman, Rettinger, Shah, & Hegarty, 2001).

This was done because a fixed task order is optimal for detecting correlations (e.g. Carlson & Moses, 2001; Carlson et al., 2004; Friedman & Miyake, 2004; Miyake et al., 2000; Miyake et al., 2001). To check whether the performance on tasks influenced one another, perhaps due to there being a finite resource for executive components (Muraven & Baumeister, 2000), participants completed the go/no-go task at the start and end of each session, and results were compared. These showed no difference, suggesting that fatigue and/or influence of similar tasks on performance did not occur.

The overall task order was as follows:

Go/no-go task—L1 VPT (ToM)—shape-matching task—go/no-go task (response inhibition)^3^—stop-signal task—director task (ToM)—go/no-go (picture) task. The tasks were split into two sessions. The first session lasted for approximately 70 minutes and consisted of the go/no-go, visual perspective-taking, and shape-matching tasks, as well as the repeat of the go/no-go task.^4^ The second session also lasted for approximately 90 minutes and consisted of the stop-signal, director, and go/no-go (picture)^5^ tasks. There were breaks within each task and between each task, so that participants were not tested continuously for the period of the sessions. Participants did the sessions on the same day (with a break between them) or on separate days (maximum gap between sessions was three weeks).

#### Analytical procedure

##### Data screening

The data set was checked for univariate outliers, and pairwise plots examined for any heteroscedasticity. Multivariate outliers were checked as per Tabachnick and Fidell (2001), resulting in 12 participants being excluded. Variances for the dependent variables were adjusted to all be within a 10:1 ratio (Kline, 2005). The final sample for analysis consisted of 142 participants (*M*_age_ = 21.76 years, *SD*_age_ = 4.37 years), with 28 males. This resulted in a total of 1,132 data points.

After variance adjustment and transformations, the final descriptive statistics for the dependent and independent variables were as shown in Table 1.

**Table 1:** Final task descriptives

Measure	*M* (*SD*)	Variance	Reliability^a^
Shape matching (ms) (square root transformation)	21.56 (4.88)	23.77	.92
Go/no-go (FAR)	4.28 (2.12)	4.51	.93
Go/no-go (picture) (FAR; Bird/Mammal)^b^	6.17 (3.05)	9.28	.74
Stop signal (SSRT) (log 10 transformation)	16.33 (6.74)	45.52	.99
Director task (errors) (log 10 transformation)			
Ambiguous	2.34 (2.41)	5.79	.59
Relational	2.75 (2.66)	7.07	.83
Visual perspective indices (IES)			
Conflict	3.62 (2.25)	5.06	.85
Focus	−.07 (2.13)	4.55	.88

^a^Split-half reliabilities

^b^As FAR for the bird and mammal trials were significantly correlated and followed the same pattern, they were collapsed to form a single FAR for the go/no-go (picture) task

An initial correlation matrix between the variables is shown in Table 2. For the inhibitory control tasks, positive correlations were shown between the shape-matching and stop-signal tasks, the go/no-go and go/no-go (picture) tasks and the go/no-go and stop-signal tasks. While these are relatively low, this is common for tasks measuring inhibition (e.g., Friedman & Miyake, 2004) and may reflect task impurity. A positive correlation was shown between the director task variables, and a negative one between the L1 VPT variables. the shape-matching task had positive correlations with both the director (relational) variable, while the go/no-go (picture) task had positive correlations with both measures of the director task. The stop-signal task also had a positive correlation with the L1 VPT (conflict) variable.

**Table 2:** Correlation matrix between variables

	Shape matching	Go/no-go	Go/no-go (picture)	Stop Signal	Director task (ambiguous)	Director task (relational)	Visual perspective (conflict)
Go/no-go	−.04	–					
Go/no-go (picture)	.00	.23**	–				
Stop signal	.15	.18*	.08	–			
Director (ambiguous)	.09	.00	.34**	.02	–		
Director (relational)	.29**	.01	.25**	.05	.65**	–	
Visual perspective (conflict)	.06	.14	.00	.24**	−.03	−.04	–
Visual perspective (focus)	−.02	.08	−.04	.02	.02	−.03	−.18*

*p < .05. **p < .01

#### Models

Analyses were carried out using AMOS 25. Path analysis aims to arrive at the most parsimonious model that explains the underlying data and does not significantly differ from it. Increasing the number of parameters in a model tends to improve fit, but necessarily decreases parsimony. The best model optimizes fit and parsimony.

**Fig. 1:**
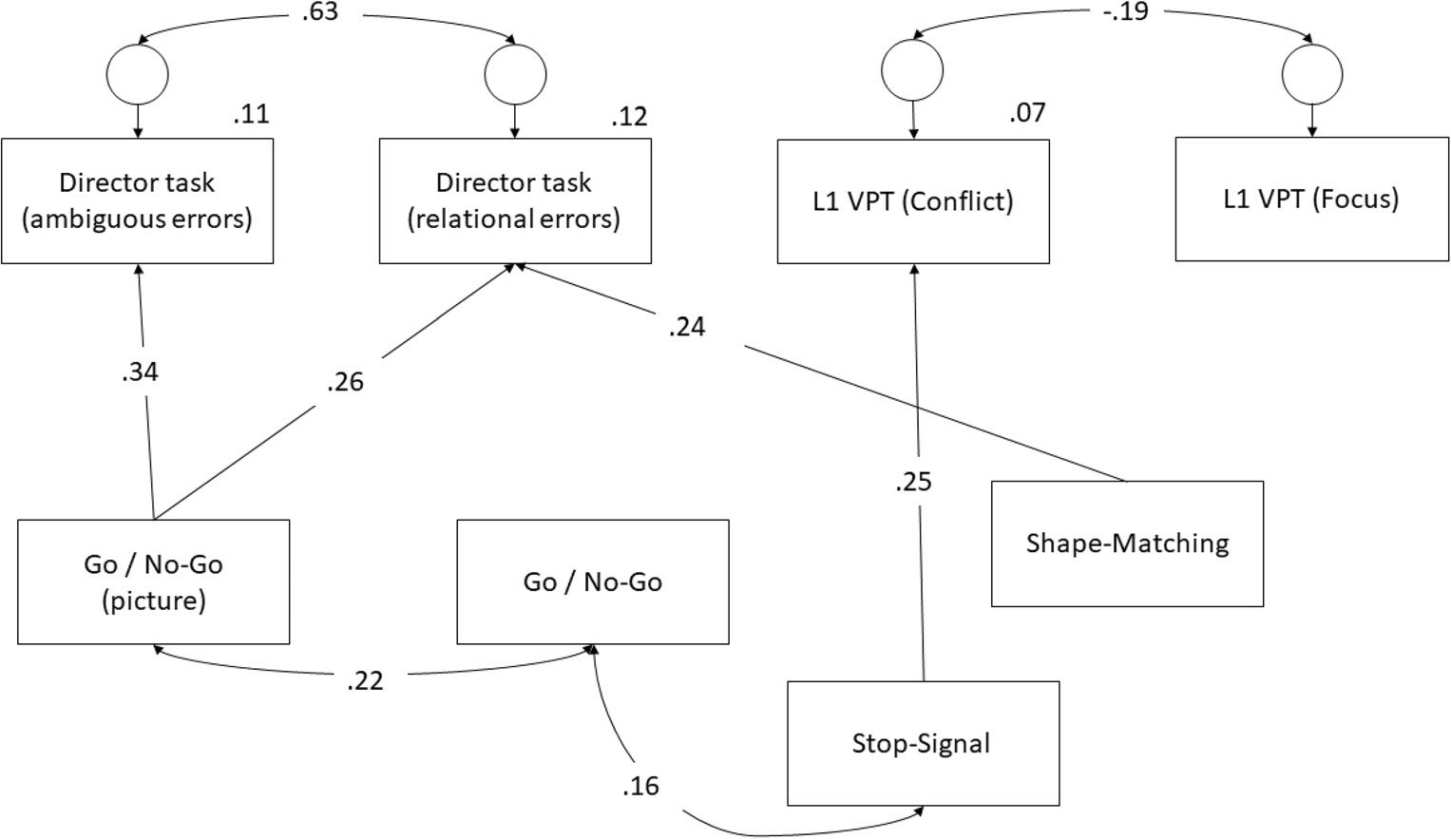
Model 3 (standardized coefficients)

While the correlation matrix suggests that for the L1 VPT task, there was only a relationship between inhibitory control and the conflict index, in order to test the hypothesis that inhibitory control accounts for self–other perspective interference and egocentrism, paths from all inhibitory control tasks to all ToM measures were included in Model 1 (see Fig. 15, Appendix B). In the second model, all nonsignificant paths were removed (see Fig. 16, Appendix B). In Model 3, covariances between the inhibitory control variables that were suggested by modification indices were added. This resulted in the final model (Fig. 1) which outlines paths from differing inhibitory control variables to both director task variables and the conflict index of the L1 VPT task. This model also evidenced relationships between the inhibitory control variables. Due to the sample size and number of parameters, Bollen–Stine bootstrap analyses were conducted to more robustly assess model fit.

## Results

Full descriptions, figures, and tables for Models 1 and 2 are shown in Appendix B and described in brief below.

### Initial model

No relationships were found between the go/no-go or stop-signal tasks and either of the director task variables. The go/no-go (picture) task predicted performance on both ambiguous and relational variables of the director task, while the shape-matching task only predicted performance on the relational variable. The stop-signal task predicted performance on the L1 VPT conflict index, and no tasks predicted performance on the L1 VPT focus index.

### Model 2

Nonsignificant paths were removed, resulting in only the paths described in the initial model. Model comparisons suggested that removing these paths did not significantly affect the overall model fit, χ^2^(12) = 6.29, *p* = .90.

### Final model (Model 3)

For Model 3, covariances between the go/no-go and go/no-go (picture) tasks and between the go/no-go and the stop-signal (SST) task were added (see Fig. 1). This significantly improved the model fit, *χ*^2^(2) = 11.38 *p* < .01.

The parameter estimates in Table 3 show that approximately 6% of the variance in the conflict index was explained by the model, with around 11%–12% of the variance of the director task (for both ambiguous and relational DVs) explained.

**Table 3:** Parameter estimates for final model (Model 3)

Path	Unstandardized estimate (*SE*)	Standardized estimate
Shape matching → Director (relational)	.13 (.03)	.24***
Go/no-go → Director (relational)		
Go/no-go (picture) → Director (relational)	.22 (.07)	.26***
SST → Director (relational)		
Shape matching → Director (ambiguous)		
Go/no-go → Director (ambiguous)		
Go/no-go (picture) → Director (ambiguous)	.26 (.06)	.34***
SST → Director (ambiguous)		
SST → L1 VPT conflict)	.08 (.03)	.25***
Covariances	Unstandardized estimate (*SE*)	Standardized estimate
Director (relational) ↔ Director (ambiguous)	3.47 (.55)	.63***
L1 VPT (conflict) ↔ L1 VPT (focus)	−.89 (.40)	−.19***
Go/no-go ↔ SST	2.29 (1.18)	.16*
Go/no-go ↔ Go/no-go (picture)	1.38 (.55)	.22*
Variances	Estimate (*SE*)	
Shape matching	23.60 (2.81)***	
Go/no-go	4.46 (.53)***	
Go/no-go (picture)	9.22 (1.10)***	
SST	45.20 (5.38)***	
e1 (director (relational))	5.99 (.72)***	
e2 (director (ambiguous))	5.10 (.61)***	
e3 (L1 VPT (conflict))	4.51 (.54)***	
e4 (L1 VPT (focus))	4.73 (.56)***	
Squared multiple correlations		
Director (relational)	.12	
Director (ambiguous)	.11	
L1 VPT (conflict)	.06	
L1 VPT (focus)	.00	

*p < .05. **p < .01. ***p < .001

The model parameters for the final model (Model 3; Table 4) show that the fit was excellent.

**Table 4:** Model fit parameters

	CMIN	*df*	*p*	NFI	CFI	AIC	RMSEA	Low	High	Bollen–Stine Bootstrap *p*
Model 1	17.35	10	.07	.88	.94	69.35	.07	.00	.13	.14
Model 2	23.64	22	.37	.84	.99	51.64	.02	.00	.08	.36
Model 3	12.26	20	.91	.92	1.00	44.26	.00	.00	.03	.87

A power analysis was conducted on the final model fit values and sample size using Preacher and Coffman’s (2006) power and sample size calculator for RMSEA. This suggests that the current sample size has a power of .92 to detect whether this was a poor fitting model.^6^ Testing whether the sample size was sufficient for the final model showed that an ideal sample size should be 107 (with .10 as the RMSEA value for a poor fit; Kenny, 2014,7). Kline (2005) suggests a 10:1 ratio for free parameters as a realistic figure, which gives an estimated sample size of 80 for the final model. The current sample size is therefore appropriate for the current analyses.

## Discussion

In the largest study to date, this research examines individual differences in adults’ performance on two ToM tasks, with a particular focus on their ability to manage interference between self and other perspectives. Since this individual difference approach is relatively novel in relation to ToM in adult participants, it is noteworthy that two widely used tasks—the director task (Keysar et al., 2003) and the Level 1 visual perspective-taking task (Samson et al., 2010) each showed reliable measurement characteristics. Moreover, performance on the two minor trial variants within the director task was strongly correlated. For the L1 VPT task, the relationship between focus and conflict indices was negative, suggesting that those better at handling conflicting perspectives were more likely to be better at taking the “other” perspective.^8^

Against this background of successful measurement, there was a striking lack of any relationship between indices of these effects in the director task and L1 VPT. There was no evidence that the need to control interference between self and other perspectives drew upon a common process in these two tasks (no support for Prediction 1), whether that is a common process specific to self–other control (Bardi et al., 2017) or a common process that relies on domain general executive control (Bull et al., 2008; German & Hehman, 2006; McKinnon & Moscovitch, 2007). Self–other interference in the two ToM tasks was correlated with measures of domain-general executive control (support for Prediction 2). However, each ToM task correlated with different executive control tasks. Since no relationship was observed between measures of self–other control, we could not examine whether such a relationship would be mediated by domain-general executive control (Prediction 3).

These results are surprising in light of the convergent evidence presented in the introduction suggesting that self–other interference and egocentrism are very commonly reported phenomena across multiple ToM tasks and other social tasks. Debate has mainly concerned whether the process for handling such interference is specific to self–other control, or domain general (e.g., Cook, 2014; Happé, Cook, & Bird, 2017; Hartwright et al., 2016). The underlying assumption that we are studying one phenomenon is usually taken for granted. Of course, we must be cautious about rejecting this assumption, particularly on the basis of the absence of predicted effects. In what follows, we first consider whether our tasks were suitable for use as *measures* of individual differences in self–other interference. We next consider whether resolution of self–other interference is a limiting step for healthy adults. Finally, we consider whether self–other interference might in fact be a family of phenomena. Given the currently limited evidence on individual differences in ToM in adults, we hope this discussion will both help interpretation of our findings and guide badly needed further work on this topic.

### Limitations in ToM measures?

Both the Level 1 visual perspective-taking task and the director task generated the anticipated self–other interference effects and measured individual differences in performance reliably. However, this does not entail that they reliably measured individual variability in self–other interference (we also note that while task reliabilities were generally high, < .7, the reliability for ambiguous errors in the director task was .59). It is important to acknowledge that both tasks were devised as laboratory platforms to investigate egocentrism and self–other interference, primarily through manipulation of task conditions that might affect performance. As recently discussed in the context of individual differences in executive control (Friedman & Miyake, 2017; Hedge, Powell, & Sumner, 2018), such laboratory tasks are typically developed to minimize individual differences between the performance of participants, in order to maximize the chances of observing between-condition effects.

In response to such limitations, a small number of other tasks have been specifically designed to quantify individual differences in ToM (see Apperly, 2012). However, these tasks generate variance by requiring more subtle or complicated judgements, and in doing so become more opaque with respect to the underlying processes contributing to variance (e.g., Apperly, 2010). A significant objective for future work is therefore to devise new tasks that maximize individual differences, not just in overall “scores,” but in theoretically motivated processes related to ToM, such as self–other interference (or perhaps other parameters related to minds, rather than representing mental states; Conway, Catmur, & Bird, 2019). With this important caveat in mind, we nevertheless note that both the L1 VPT and the director task have previously shown evidence of construct validity, through correlations with well-established measures of individual differences relevant to social functioning (focus scores for L1 VPT correlate with the Interpersonal Reactivity Index; Bukowski & Samson, 2017; errors on the director task correlate with traits related to both autism and schizophrenia; Abu-Akel et al., 2015). We consequently believe the present findings are interpretable, given appropriate caution.

A further question is whether these tasks actually measure ToM at all, since recent evidence suggests that the processing they entail may not be domain specific to ToM (e.g., Santiesteban et al., 2014; Santiesteban, Kaur, Bird, & Catmur, 2017; Santiesteban, Shah, White, Bird, & Heyes, 2015). There has been longstanding discussion about the extent to which ToM relies upon distinctive cognitive processes. One position is that reasoning about mental states makes unique cognitive demands (e.g., Leslie, 1987) that are met by a domain-specific module (e.g., Baron-Cohen, 1995; Leslie, Friedman, & German, 2004). A different, and widely held position is that ToM is more domain general, making similar cognitive demands to a much broader category of reasoning problems, including reasoning about signs, words and pictures, as well as counterfactual thinking (Perner & Leahy, 2016; Riggs, Peterson, Robinson, & Mitchell, 1998; Roessler & Perner, 2013). Both positions are compatible with a significant role for executive processes in the performance of ToM tasks, but the domain-general account also allows that executive processes contribute directly to representing or reasoning about mental states. Researchers have long sought evidence of domain specificity by comparing behavioral performance when participants reason about mental states versus pictures, signs, or other nonmental perspectives, with relatively few results supporting domain specificity. For example, while children with autism perform worse on false-belief tasks than on comparable false-photograph tasks (Leslie & Thiass, 1992), they perform at similar levels on false-belief and false-sign tasks (Iao & Leekam, 2014). Typically developing children’s performance on false-belief tasks is closely correlated with performance on false-sign tasks (Leekam, Perner, Healey, & Sewell, 2008) as well as synonym-judgement tasks (Doherty & Perner, 1998), all of which require reasoning about metarepresentations, but not mental states (Perner & Leahy, 2016). In response to such results, it has been argued that false-belief tasks (and ToM more generally) make demands that are exacting and meaningful in terms of understanding patterns of performance in children and adults, but these demands are not domain specific to ToM (e.g., Apperly, 2010). To our knowledge, nobody has used such results to argue that false-belief tasks do not entail representing mental states. One reason for this might be that participants in these tasks are often directly requested to reason about mental states.

Specifically in relation to the L1 VPT and director tasks used in the current study, recent work that has tested the domain specificity of these effects and found evidence that similar effects are obtained when the avatar is replaced with an arrow and when the director is replaced with a camera the view of which conditionalizes their responses in the same way as the director (e.g., Santiesteban et al., 2014; Santiesteban et al., 2017; Santiesteban et al., 2015). These results fail to find support for the domain specificity of the effects in the L1 VPT and director tasks, but as illustrated above, there is no necessary link between ToM and domain specificity in accounts of ToM, and plenty of reasons for thinking that domain-general executive functions play central roles in enabling ToM. Moreover, while it cannot be ruled out that participants ignored instructions to think about the perspectives of the avatar and the director, we see no evidence for this (e.g., Apperly et al., 2010), and no reason why participants would have behaved in this way.

The concern that participants may not have been representing mental states at all has most force in the “self” condition of the L1 VPT task in which participants were not explicitly asked to judge the avatar’s perspective. Instead putatively “implicit perspective taking” is inferred from interference effects on participants’ judgements of their own perspective. In this case it is informative to view results from the “self” and “other” trials separately (see Appendix C). These analyses find that the “altercentric interference” effect for self trials is not related to any of the executive tasks in the present study, but it is related to “egocentric interference” on other trials of the L1 VPT task. Of course this evidence does not demonstrate that perspective-taking *is* occurring, and different patterns may have been obtained with different inhibition tasks. Nor would a significant correlation have demonstrated that perspective taking was *not* occurring; it would not be surprising if the magnitude of interference from the avatar’s perspective varied as a function of an individual’s inhibitory capacity. However, as it stands, the present study provides no evidence that altercentric interference reflects domain general inhibition and not perspective taking, which is the alternative hypothesis that has sometimes been proposed for this task (e.g., Heyes, 2014).

### Is self–other control a limiting step for healthy adults?

Existing evidence suggests that self–other interference effects are particularly large in children (who have limited executive control; Steinbeis, 2016), in individuals with brain injury that has affected their capacity for executive control (e.g., Samson et al., 2005; Samson et al., 2015), and in healthy adults under cognitive load (e.g., Qureshi et al., 2010). Healthy adults without cognitive load also show self–other interference effects, and associated neural correlates (Cook, 2014; Steinbeis, 2016). However, the fact that healthy adults must resolve self–other interference in a given task does not mean that doing so is a limiting factor on their performance such that individuals might vary in their capacity to resolve this interference successfully. On this interpretation, there may be a common underlying process involved in self–other control across different tasks, but since all healthy adults could meet this need reliably it would not contribute to individual differences in performance that would correlate between tasks. Correlations might only emerge if the ToM tasks placed sufficiently large demands on self–other control that healthy adults did vary in their capacity to meet these demands. Alternatively, the correlations that we did observe between self–other interference effects and measures of domain-general executive function could suggest that our tasks were indeed sufficiently taxing to generate at least some individual differences. However, these correlations accounted for only a small proportion of the variance in performance, and the fact that different executive tasks correlated with each ToM task calls into further question the proposition of a common self–other factor between the ToM tasks.

### Self–other control and egocentrism may be a “family” of phenomena that place different executive demands

It is a parsimonious hypothesis that all self–other interference effects arise from a common underlying process, but this may not be the best explanation for these phenomena. The need to distinguish mental states related to self and other surely is a core feature of ToM tasks, but it has been suggested that this feature can give rise to interference effects in different ways, and at different stages of processing (e.g., Apperly, 2010). This account would predict a “family” of self–other interference phenomena that resemble each other at a general level of description, but are not necessarily correlated because different demands are made at different stages of processing. For example, in the L1 VPT task participants must both *calculate* the perspectives of self and other and *select* the correct response based on one or other of these perspectives (Qureshi et al., 2010). Evidence from event-related potentials suggests that perspective calculation occurs first and involves posterior cortex (putatively temporoparietal junction), while selection occurs later and involves the frontal cortex (putatively the right inferior frontal gyrus; McCleery et al., 2011). Importantly, *both* calculation and selection ERP effects are influenced by self–other consistency, but participants’ response times and errors on this task appear to reflect the effect of self–other consistency on selection of a response according to the perspective required for that trial. The correlation observed in the present study between the conflict index from the L1 VPT task and the stop-signal task suggests that this selection may occur very late in processing, perhaps after responses according to both self and other perspectives have already been initiated.

In contrast, while the director task requires Level 1 visual perspective taking, it additionally requires that information about the director’s perspective is integrated with his or her instructions about which objects to move. Studies of eye movements suggest that participants may process the director’s perspective in anticipation of his or her instruction, and that egocentric eye movements and errors originate in difficulty with the subsequent process of integrating this information with his instruction (Barr, 2008). The proposal that egocentric effects in the director task have a different functional origin from self–other interference effects in the L1 VPT task may explain why these effects did not, themselves, correlate, and why different domain-general executive tasks were correlated with each effect.^9^

## Conclusion

From the largest exploration of individual differences in in ToM and inhibitory control to date, we found that self–other interference effects in L1 VPT and director task were dissociable. Individual differences in performance on the two tasks were unrelated, and differences in performance on each task were related to different tests of executive function. Both findings converge with recent evidence from individual differences in perspective taking during psycholinguistic tasks (Ryskin et al., 2015). We suggest that self–other differences are part of the essence of ToM tasks, but that they give rise to interference effects in a variety of ways, at different stages of processing, resulting in a variety of requirements for executive control processes. We also suggest that the requirement to resolve self–other interference may not be the only, or even the most significant, limiting step for healthy adults’ performance on typical tasks. Future work is needed to understand whether self–other interference sometimes places a higher burden for adults, and also to understand other factors that may limit adults’ ToM performance, and so may explain individual differences in social ability.

## Electronic supplementary material

ESM 1 (DOCX 495 kb)

ESM 2 (DOCX 221 kb)

ESM 3 (DOCX 333 kb)

## Footnotes

1 The original design selected two sets of three inhibitory control tasks as indicators for two latent variables for cognitive and response inhibition. However, the covariance structure did not support this theoretically motivated distinction between latent variables, and so tasks with adequate measurement properties were instead utilized as individual predictors.

2 Use of IES is not recommended if speed–accuracy trade-offs are shown (indicated by no positive correlation between response times and error rates) and if the average error rate is above .10 (see Bukowski & Samson, 2017). The correlation between response time and error rate was positive, *r*(142) = .20, *p* < .01, and the mean error rate across conditions was 0.06 (standard deviation = 0.07), meaning both recommendations for using IES were met.

3 This was a repeat of the first task in the session to compare performance at the start to the end of the session to see the effect of a.) fatigue on performance, and b.) the possibility of the reduction in the resource of self-control affecting performance by the end of the session (Muraven & Baumeister, 2000).

4 And the Simon Task which was not included in the final analyses

5 And Cued Recall task which was also not included in the final analyses

6 Observed power = .8, *df* = 20, *n* =142, null RMSEA = .01 (excellent fit), alternative RMSEA = .00 (final model value).

7 Observed power = .8, *df* = 20, null RMSEA = .01, alternative RMSEA = .10.

8 Lower conflict index indicates better at dealing with conflicting perspectives; higher focus index suggests relatively better performance when taking “other” perspective compared with self-perspective.

9 A more specific discussion of the relationship between the individual executive tasks, and their relationships with the ToM tasks is in section A4 of Appendix A.
