# Supplementary material for: Does interference between self and other perspectives in theory of mind tasks reflect a common underlying process? Evidence from individual differences in theory of mind and inhibitory control
Source: Psychon Bull Rev. 2019 Aug 19;27(1):178–90. doi: 10.3758/s13423-019-01656-z (PMC7000534; doi:10.3758/s13423-019-01656-z)
Supplement: Supplementary file 1 — (DOCX 495 kb) [file 13423_2019_1656_MOESM1_ESM.docx]

**Appendix A1**

**Inhibitory control task selection and predictions**

The classification and choice of inhibitory control tasks was based on Friedman and Miyake (2004). Their initial classifications were designed to measure the different stages of the informational processing of inhibition. This starts with resistance to distracter interference (selecting relevant information and ignoring irrelevant information), followed by resistance to proactive interference (once information has entered working memory), and, finally, prepotent response inhibition (select relevant responses and resist incorrect ones). They found that resistance to distracter interference and prepotent response inhibition loaded on the same latent variable (defined as response-distracter inhibition). As such, it was concluded that response-distracter inhibition and resistance to proactive interference are distinct components of inhibition (Friedman & Miyake, 2004). Our tasks we used were therefore originally chosen with the objective of identifying latent variables for these two components and then assessing their relationship to ToM.

In Friedman and Miyake (2004) response-distracter inhibition tasks included the go/no-go task (though these also include some requirement for response selection), stop-signal task, and antisaccade tasks. Due to laboratory infrastructure, the antisaccade task could not be used, so we selected a variant go/no-go task where the go and no-go judgements were based on semantic and orthographic information, as opposed to perceptual information. This follows the principle that latent variables are best estimated using tasks that differ in as many respects as possible other than the hypothesized underlying common process. From the tasks that Friedman and Miyake (2004) found to load on, on the latent variable for resistance to proactive interference, we selected the cued-recall task, Simon task, and shape-matching tasks.

However, initial data screening and confirmatory factor analyses showed that two of the chosen tasks (Simon and cued-recall tasks) showed no relationship between any of the other tasks and low reliability, respectively. We therefore had to exclude these tasks from further analyses and follow an alternative analytical approach of using the remaining tasks as individual predictors.

Specific predictions about potential relationships between the individual inhibitory predictors and the ToM tasks are as follows:

1. The director task involves the participant avoiding interference from their own perspective when integrating information about the director’s perspective with his or her instructions, and also the need to avoid selecting a distracting incorrect response (Barr, 2008). This leads to the expectation of a role for both response-distracter inhibition and resistance to proactive interference, and therefore potential relationships with any of the selected inhibition tasks.
2. The L1 VPT conflict index relates to interference between self–other perspectives, and literature suggests that participant errors and response times reflect the effect of self–other consistency on selection of a response (Apperly, 2010; McCleery, Surtees, Graham, Richards, & Apperly, 2011; Qureshi, Apperly, & Samson, 2010). This suggests a functional role for response-distractor inhibition, and so we predicted a relationship between L1 VPT conflict index and inhibition tasks involving response-distractor inhibition. The focus index represents relative performance on judgments that relate to the self (vs. other). As such, we did not expect to see a relationship with the focus index and inhibitory control tasks.

**Appendix A2**

**Task methodology (further details)**

***Shape-matching task***

The shapes used were taken from DeSchepper and Treisman (1996); eight each of green (target), red (distracter), and white (matching) shapes. The task was simplified to measure resistance to distracters and resistance to inappropriate responses, rather than negative priming as in DeSchepper and Treisman (1996). This was done by presenting the distracter shapes at the same time as target shapes in the experimental trials, which requires participants to actively keep the distracters from interfering with processing of the target shapes (Endo, Saiki, & Sato, 2001).

The shapes measured 135 × 124 pixels and were presented on the left (green/red shapes) and right (white shape) of a 300 × 300-pixel black square. The number of times each type of shape occurred was balanced across the task. Across the total of 112 trials (56 distracter trials and 56 no-distracter trials), the number of times each shape occurred was balanced in four separate blocks of 28 trials. Within these, the number of matching (green = white) and nonmatching (red = white) stimuli were also balanced.

Participants were instructed to decide whether the green (target) shape matched the white shape. They were told to ignore the red (distracter) shape when it was present. Each trial had the following procedure: The participants were presented with a READY prompt, to which they had to respond to in order to proceed. A blank screen was then shown for 1,100 ms, followed by a fixation point for another 500 ms. The shapes were then presented until the participant responded. Then another blank screen was shown for 100 ms, upon which the READY prompt appeared for the start of the next trial. Images of the trials are shown in Fig. 2:

| **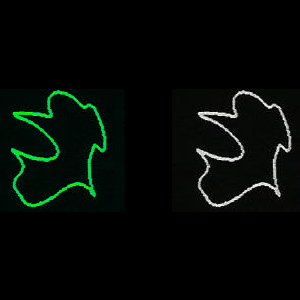** | **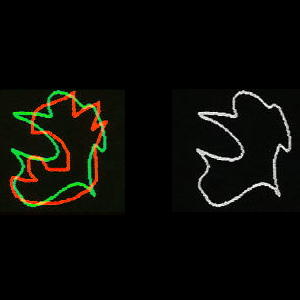** |
| --- | --- |
| 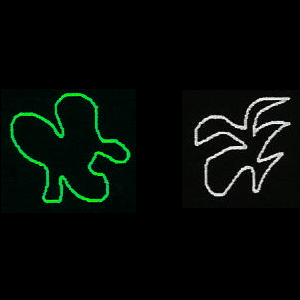 | 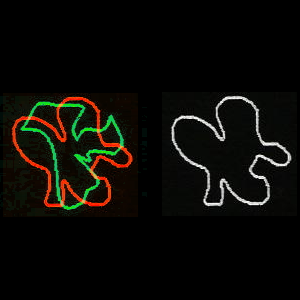 |

Fig. 2 Trial images: Top left = no distracter, match. Top right = distracter, match. Bottom left = no distracter, no match. Bottom right = distracter, no match

***Cued-recall task***

The task was designed to measure proactive interference and was based on the study of Tolan and Tehan (1999). Participants were presented with either one block (filler trials) or two blocks (control and experimental trials) of four serially presented words (one per 1,000 ms). In all trials this was followed by a numerical magnitude judgment task as a distraction.^[[1]](#footnote-1)^ Participants were then presented with a cue word to vocally recall a word from the only block for filler trials or from the second block (control and experimental trials). The cue word was a category word related to the target word in the second block, but also to a foil word in the first block of experimental trials (there was no foil word in control trials). The foil words were always a more common instance of the category given by the cue word (than the target words), so increasing proactive interference. An example experimental trial is shown in Figure 3.

Block 1 (read aloud):


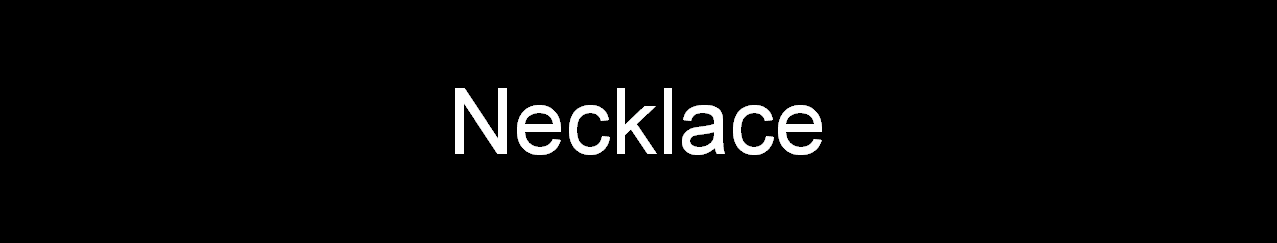

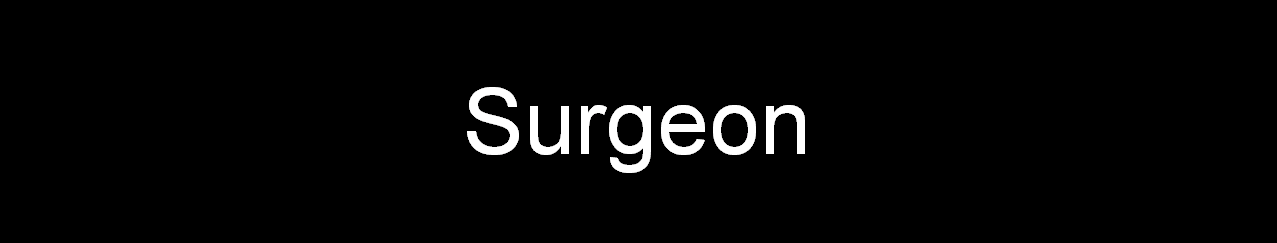

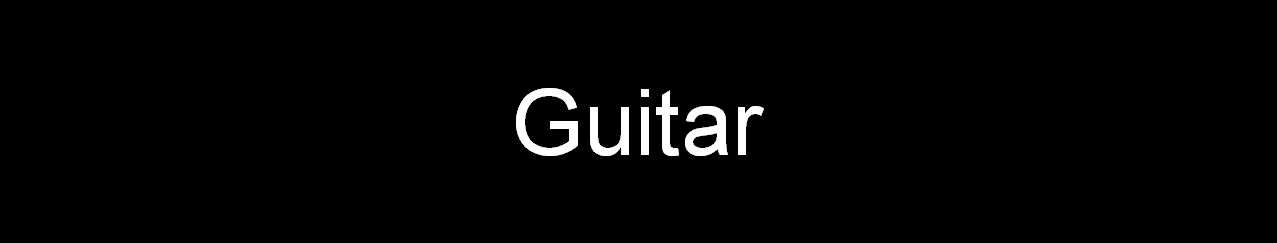

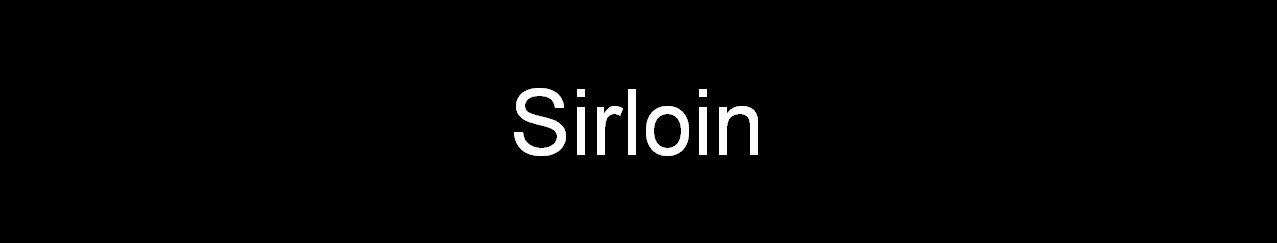


Block 2 (read silently):


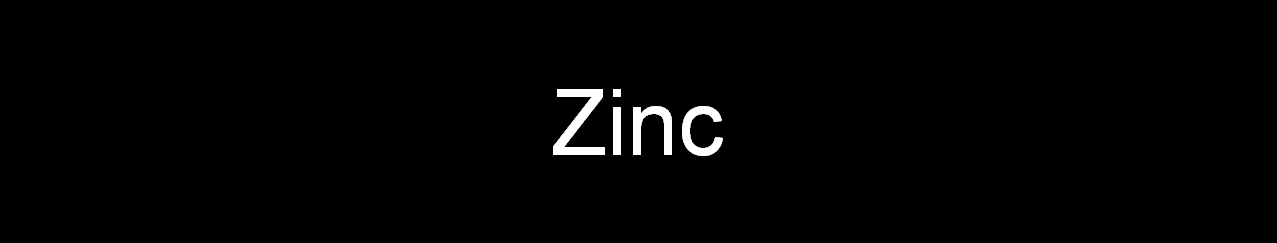

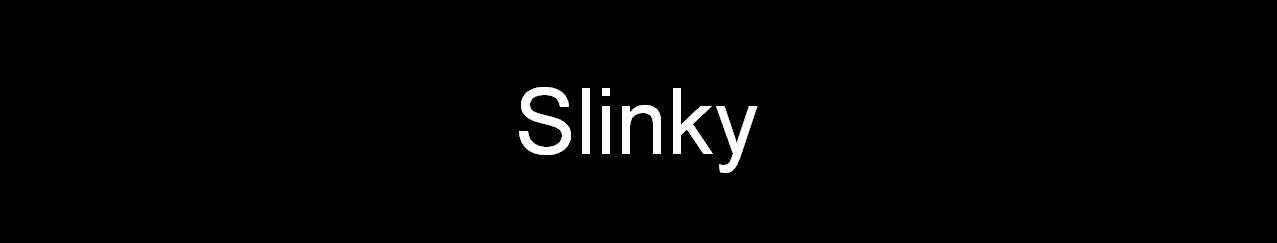

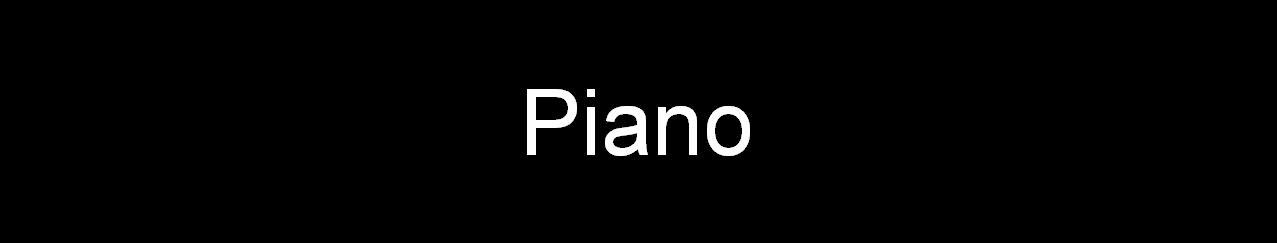

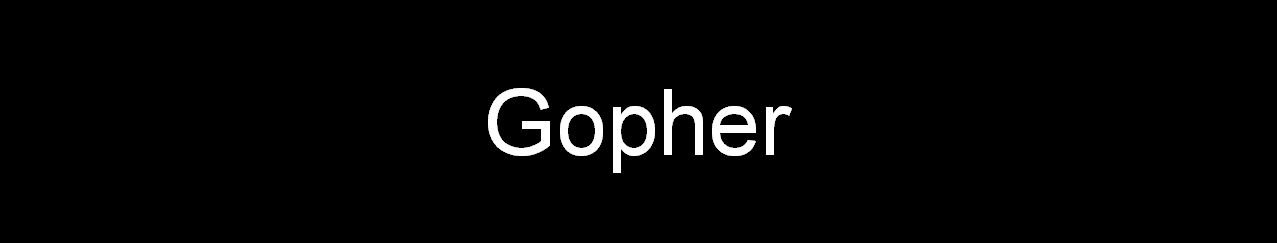


Distracter task

Cue:


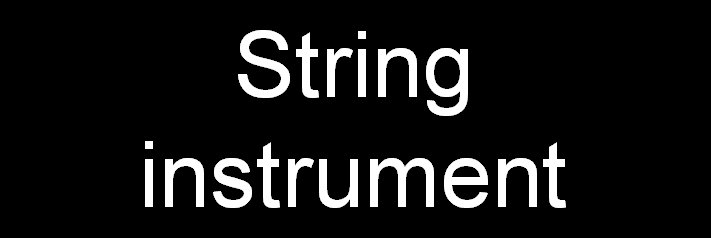


Fig. 3 Cued-recall experimental trial (for the purposes of illustration, the blue border indicates foil word, and the red border indicates target word; these borders were not present in experimental stimuli). (Color figure online)

The experiment consisted of 10 one-block filler trials and 40 two-block trials, the latter consisting of 20 control and 20 experimental trials. To increase interference, the ALOUD block always came before the SILENT block. This was followed by the distracter task and then the cue word. The dependent measure was the number of correct control trials minus the number of correct interference trials. This was chosen because the number of correct control trials was taken to be the measure of the participants’ general recall ability (dealing with the interference caused by the mixed modality (reading aloud vs. silently), and the distracter task). The interference trials additionally had pro-active interference from the foil word in the first block. The measure therefore assessed participants’ ability to deal with proactive interference while taking into account their general recall ability.

***Simon task***

The task was based on Peterson et al. (2002). Participants were shown horizontally orientated arrows (left or right), presented on the left or right of a central fixation point. Participants were instructed to respond according to the orientation of the arrows, not the position. As the arrow position is visually more salient than the arrow orientation, responding to the orientation requires the inhibition of any response to the position. The majority of trials were congruent for orientation and position (328 congruent, 82 incongruent), with left and right positions and orientation balanced equally across four blocks of 102 trials for a total of 408 trials.

Each trial began with a fixation point for 200 ms, followed by an arrow for a maximum of 1,300 ms or until a response was made. The difference between response times to accurate incongruent and congruent trials was taken to be a measure of any inhibitory processing cost present only in the incongruent trials (any processing cost common to the incongruent and congruent trials would be accounted for).

***Go/no-go task***

The experiment consisted a total of 520 trials across eight blocks of 65 experimental trials (each block also had an additional five trials at the start; these were eliminated in analyses as practice trials). There were either eight or 10 no-go (K) trials (and a corresponding 57 or 55 go trials) in each block. Each letter was presented centrally on a black screen.

Participants viewed a series of letters presented on a computer screen to which they had to respond by pressing the space bar, except for the letter *K*. If the participant responded incorrectly to the letter *K*, a tone would be heard for 250 ms. Each trial consisted of an initial fixation cross for 250 ms, followed by a letter. Each letter remained on-screen for 500 ms, or until the participant responded.

***Go/no-go (picture task)***

This task was based on the study of Schmitt, Münte, and Kutas (2000). Participants were presented with images of either birds or mammals (there was a familiarization procedure prior to the task^[[2]](#footnote-2)^). Participants responded by pressing the *Q* key if a bird was shown, and the *P* key if the image was of a mammal. If the name of either began with a vowel, they were instructed not to respond. This task creates a go/no-go situation because an initial go decision is based on the rapidly available semantic information (bird/mammal category), which must then be cancelled or allowed based on later-available phonological or orthographic information (van Turennout, Hagoort, & Brown, 1997). The majority of images were go trials (36 each for birds and mammals, compared with nine no-go trials for each), repeated twice and balanced across eight blocks for a total of 180 trials. The first three trials were removed from further analyses as practice trials.

During the experiment, after an initial fixation cross for 250 ms, the images were presented for 750 ms, or until the participant responded. If the participant responded incorrectly, a tone was heard for 250 ms. The images were scaled to 500 × 500 pixels.

***Stop-signal task***

A total of 256 trials across eight blocks were presented to participants, consisting of either an “X” or “O.” After an initial fixation cross for 500 ms, the stimuli were shown on-screen for 1,000 ms or until the participant responded, followed by a blank screen for 2,000 ms (or again until the participant responded). Sixty-four of the trials were stop trials, where a tone was heard after a variable delay, with the remaining being go trials, all balanced across blocks. Participants were instructed not to respond if they heard the tone, and the stop-signal delay started at 250 ms after stimuli presentation and then was followed a dynamic tracking procedure (+50 ms if they responded correctly, and −50 ms if incorrectly). Letters were presented centrally on a 400 × 300 pixel black screen.

***L1 VPT task***

There were a total of 96 matching trials: 48 trials in which participants were asked to verify their own perspective (with 24 consistent perspective trials and 24 inconsistent perspective trials) and 48 trials in which participants were asked verify the avatar’s perspective (with 24 consistent perspective trials and 24 inconsistent perspective trials). In addition to these 96 test trials, 96 mismatching filler trials were included (with the same distribution as for test trials) as well as 16 additional antistrategy filler trials (eight matching and eight mismatching in which no circles were pinned on the wall). The 208 items were split into four blocks of 52 items. Each trial image measured 640 × 480 pixels, with the avatar presented centrally. Between zero and three dots were presented at the avatar’s eye level on neither, both, or either wall.

Before being shown the image, participants were cued with a verbal perspective description that informed them which perspective to take (“you” vs. “he”/”she”) and which perspective content to verify (i.e., a number of dots visible, between zero and three). They were then asked to decide whether the cue information matched the relevant content shown in the image. In half of the trials, the cue matched the image, and on the other half, the cue did not match the image. An initial fixation cross was on-screen for 750 ms, followed by the cues, which were also shown for 750 ms. The gap in between fixation and cues was 500 ms. The stimuli picture remained on-screen until the participant responded. If no response was received within 2,000 ms of the picture being on-screen, the trial was recorded as no response. Trial conditions are shown in Fig. 4:

Fig. 4 L1 VPT trial conditions

***Director task***

Stimuli replicated those in Apperly et al. (2010). A total of 32 different grid arrays were used, each with 16 slots (4 × 4 grid). In each grid, five slots were occluded from the view of the director (male figure with a male voice), with the remaining 11 visible to the participant and the director. These were arranged in one of four different patterns, sized at 720 × 540 pixels. Each grid contained eight items, created from simple cartoon images. There were 128 instructions used across the experiment, with between three and five for each grid. Two practice grids were also used with the same layout.

Sixteen of the grids were “experimental” grids, with one critical instruction that could refer to an object in an occluded slot (if only the participant’s perspective was taken into account), or to one in a mutually visible slot (if the director’s perspective was accounted for). The position of this critical instruction varied between first and fourth in the series for each grid. Half of the experimental grids were “relational” trials, and half were “ambiguous” trials. Relational instructions followed the pattern “Move the [adjective noun] one slot left/right/up/down,” and the noun occurred on average 1,047 ms after the start of the instructions. For ambiguous trials, the instructions followed the formula “Move the [noun] one slot left/right/up/down,” and the noun occurred on average 742 ms after the instruction. All other instructions were filler items that followed the same pattern of the critical instruction, and always referred to mutually visible objects.

The remaining 16 grids were matched control grids (also half relational and ambiguous), where the item referred to in the critical trial was replaced with an item that could not be a potential referent (e.g., aeroplane for small ball). Every grid was presented for 5,000 ms before instructions began, and these were then given at 5,000-ms intervals. An example grid is shown in Fig. 5.

**Fig. 5** Example grid for a relational grid of the Keysar task. Critical instruction: “Move the small ball down the slot.” In the experimental grid, the smallest ball from the participant’s viewpoint is the competitor item (golf ball), but the target item (the smallest ball from the director’s viewpoint) is the tennis ball. In the control grid, the smallest ball from the participant’s viewpoint is the target item (tennis ball), which is the same as for the director

**Appendix A3**

**Individual task results**

The data-screening process consisted of several steps. The following steps were adapted from those used by Friedman and Miyake (2004) and Oberauer (2005) in order to reduce the effect of any extreme response time values on the mean.

The critical dependent variables of the various tasks were calculated, and any values more than three standard deviations above or below the task mean were replaced by the cutoff value (of three standard deviations above or below the mean).^[[3]](#footnote-3)^ All pairwise plots were then examined for nonlinearity and heteroscedasticity. The distributions of the critical dependent variables were then checked, as were univariate outliers, and any transformations were carried out. Multivariate outliers were then checked, and finally the variance of each variable was examined and modified as per Kline (2005). Each task required different calculations for its critical dependent variable, which are all detailed below. The reliability of all the critical measures was calculated by adjusting split-half correlations using the Spearman–Brown prophecy formula (Salthouse, Siedlecki, & Krueger, 2006). Each task was first analyzed individually to establish that the expected effects were observed.

***Go/no-go***

The data were analyzed using a two-way (position; start vs. end) repeated-measures ANOVA. The dependent variable was the false-alarm rate (FAR) on no-go trials.

There was no difference in the performance of participants in the task carried out at the start of the session and the performance in the task carried out at the end of the session (see Fig. 6; *F*(1, 141) = .35, *p* = .55, η_p_^2^ = 0.02, so FAR was collapsed over position. FAR was significantly different from zero, *t*(141) = 23.53, *p* < .01.

Fig. 6 FAR by session (bars = standard deviation)

As the distribution was skewed, a square-root transformation was carried out. The reliability of the measure was excellent (.93).

***Go/no-go (picture)***

A repeated-measures ANOVA was conducted, with image type (bird vs. mammal) as the condition, and FAR as the dependent variable. There was a main effect of image, with higher FAR for bird images than for mammals, *F*(1, 141) = 9.67, *p* < .01, η_p_^2^ = .07 (see Fig. 7). However, due to the correlation between the FARs for birds and mammals suggesting similar processes were involved, they were collapsed to form a single FAR measure. The reliability of the measure was satisfactory (.74).

Fig. 7 FAR by image (bars = standard deviations)

***Stop-signal task***

The critical dependent variable was the mean response time to the go trials (correct response only) minus the mean response time to the stop trials. The mean stop signal response time (SSRT) values of the participants was similar to that found in previous literature (232 ms, *SD* = 237.09) compared with 189 ms in Aron et al. (2003).

The distribution of SSRT was extremely nonnormal. In order to normalize the distribution, the times above three standard deviations were replaced by the highest value below three standard deviations plus one (for the lowest above three standard deviations, plus two for the second lowest, etc.). This was done as described above in three waves—twice for values above three standard deviations, and once for values below three standard deviations, with two participant values being replaced (the mean SSRT after this trimming was 219 ms, *SD* = 172.41). These procedures are as described in Tabachnick and Fidell (2001). This resulted in the distribution being reasonably normal. The reliability was excellent (.99).

***Shape-matching task***

Data were trimmed by condition for each participant individually, looking at correct responses only (5.1% of the responses were incorrect). Response-time values that were more than three standard deviations away from the condition mean for a participant were replaced by the cutoff value (of three standard deviations from the condition mean). This was done for 1.41% of trials. A 2 × 2 repeated-measures ANOVA was used to examine the data. The factors were distracter (present vs. not present) and match (did the target match the green shape, yes vs. no) and the measure was the mean response time for each condition.

There was an effect of distracter, *F*(1, 141) = 122.73, *p* <.01, η_p_^2^ = .47. If the distracter shape was present, the response time was significantly longer than when the distracter shape was not present (distracter = 1296.10 ms > no distracter = 973.07 ms). There was also an effect of match, *F*(1, 141) = 25.13, *p* < .01, η_p_^2^ = .15. Participants took significantly longer to respond to trials in which the green shape did not match the white shape than to trials where they did (match = 1,093.12 ms < no match = 1,176.05 ms). There was an interaction between distraction and matching, *F*(1, 141) = 4.65, *p* < .05, η_p_^2^ = .03, as can be noted in Fig. 8. Errors were similar for all conditions (proportion of errors in distracter no-match condition = .07, in distracter match condition = .05, in no-distracter no-match condition = .04, in no-distracter match condition = .04), indicating that there was no particular trade-off between speed and accuracy.

Fig. 8 Response time by condition (bars = standard deviations)

Simple main effects showed that for both match and mismatch conditions, participants took significantly longer to respond to trials where the distracter was present (*p*s < .01). When the distracter was present, there was no difference between the match and mismatch conditions (*p* = .08), but participants took longer to respond to the mismatch condition when the distracter was not present (*p* < .01).

The critical dependent variable was the difference in mean response time between the distracter no-match condition and the no distracter match condition. The distracter no-match condition appeared to have the largest amount of interference from the distracter shape, as this shape matched the target. The no-distracter match condition had the least amount of interference, and gives a baseline measure of the response time to the simple matching process. The difference between these conditions is therefore a measure of the interference caused by the distracter shape only, which should require inhibitory control. Any values more than three standard deviations away from the mean were replaced by the cutoff value (of three standard deviations above or below the mean). The distribution of the critical shape matching task dependent variable was extremely non-normal, so underwent a log 10 transformation resulting in a normal distribution. Reliability was adequate (.66).

***Cued recall***

As the data analyzed for the cued-recall task were the number of correct trials, they were not trimmed (9.5% of the responses were incorrect). Control and interference trial performance was analyzed, using a two-way repeated-measures ANOVA. In both trial types, participants were cued to recall a target word from a list (the second of two). In interference trials, a foil word was also present (in the first list of the two). The first list of words was always read aloud, and the second set was always read silently. Before the analysis was carried out, missing values for two participants^[[4]](#footnote-4)^ on task were estimated through imputation and regression estimation procedures. The result of the estimations from these two procedures did not differ significantly, so the estimates from the imputation method using the program NORM (Schafer, 1999) were used.

Participants were more accurate in the control condition than in the interference condition, *F*(1, 141) = 191.38, *p* < .01, η_p_^2^ = .59 (see Fig. 9). This effect is probably due to the effect of the foil word in the interference trials affecting recall of the target word, as expected. The critical dependent variable was calculated as the difference between the number of correct control trials and the number of correct interference trials. The mixed modality of the presentation and distracter task were common to both trials, so this dependent variable should remove any contribution from that. The dependent variable should therefore only measure the level of interference caused by the foil word in recalling the target word, interference which should be mediated by inhibitory control. The distribution of the critical dependent measure was normal, so no transformations were required. However, the reliability of the measure was extremely low (.12), so this measure was excluded from the final analyses.

Fig. 9 Errors (*N*; bars = standard deviation)

####

#### *Simon task*

The data were trimmed by condition for each participant for correct responses only (5.3% were incorrect responses). Response time values that were more than three standard deviations away from the condition mean for a participant were replaced by the cutoff value (of three standard deviations from the condition mean). A 2 (position; left × right) × 2 (orientation; left × right) repeated-measures ANOVA was used to examine the data, with the dependent variable mean response time for correct trials.

There was no effect of the position of the arrow on the response time of participants, *F*(1, 152) = .77, *p* = .38, η_p_^2^ = .01, whilst there was a significant effect of orientation, *F*(1, 152) = 36.34, *p* ≤ .01, η_p_^2^ = .19, with responses to left-orientated arrows significantly slower than to right-orientated arrows. This may be due to the majority of the sample being right-handed participants.

There was a significant interaction between position and orientation, *F*(1, 152) = 567.16, *p* ≤ .01, η_p_^2^ = .79, as shown in Fig. 10. Simple main effects were conducted to analyze this further.

Fig. 10 Mean response times (and standard error) to differing positions and orientations of arrow

Simple main effects indicated that when the orientation and position of the arrow was congruent, response times were significantly faster, relative to when they were incongruent (all *p*s < .01). The pattern of errors showed a similar effect, with more errors made when position and orientation were incongruent (.13) compared with when they were congruent (.35), indicating no speed–accuracy trade-off.

The critical dependent variable of the Simon task was the difference between the mean response times to incongruent stimuli and congruent stimuli. The reliability of this measure was satisfactory (.92). This was taken to measure the interference between the dominant response and correct response to the incongruent stimuli, interference that should require inhibitory control to resolve. The distribution was examined, was found to be skewed, so was square-root transformed, resulting in a normal distribution.

***Theory of mind tasks***

***L1 VPT.*** Data trimming was conducted on correct responses only on the matching trials (4.78% of the data were incorrect responses). Response times that were 2.5 standard deviations away from the mean were eliminated (2.04% of the data; in line with Qureshi et al., 2010), and so were response omissions due to the time-out procedure (responses over 2,000 ms; 0.47% of the data) for a total of 3.92% of all trials. The data were analyzed using a 2 × 2 repeated-measures ANOVA, with consistency (consistent vs. inconsistent) and perspective (self vs. other) as the factors. The measure first investigated was response time in each of the conditions.

There was a main effect of consistency, *F*(1, 141) = 288.59, *p* < .01, η_p_^2^ = 0.67, with the response time in the inconsistent condition significantly higher than that in the consistent condition. There was no effect of perspective, *F*(1, 141) = .85, *p* = .36, η_p_^2^ =.01. There was a significant interaction between consistency and perspective, *F*(1, 141) = 63.52, *p* < .01, η_p_^2^ = 0.31 (see Fig. 11) that was analyzed further using simple main effects.

For both other-perspective and self-perspective judgments, response times to inconsistent trials were significantly longer than for consistent trials (*p*s < .01). For consistent trials, self-perspective judgements were significantly longer than other perspective judgement trials (*p* < .01), whereas for inconsistent trials, other perspective judgements were significantly longer than self-perspective judgements (*p* < .01)

Fig. 11 Response times (bars = standard deviation) by condition and trial

The errors (not trimmed) in each condition were examined using an identical 2 × 2 repeated-measures ANOVA. There was a main effect of consistency, *F*(1, 141) = 176.31, *p* < .01, η_p_^2^ = .56, with more errors in the inconsistent condition than in the consistent condition. There was again no effect of perspective, *F*(1, 141) = 1.09, *p* =.30, η_p_^2^ = .01. There was a significant interaction between consistency and perspective, *F*(1, 141) = 14.46, *p* < .01, η_p_^2^ = .09, investigated further using simple main effects (see Fig. 12).

For both other and self-perspective judgments, there were more errors on inconsistent trials than on consistent trials (*p*s < .01). For consistent trials, there were more errors for self-perspective judgements than for other perspective judgement trials (*p* < .01), whereas for inconsistent trials, there were more errors for other perspective judgements than for self-perspective judgements (*p* < .05).

Fig. 12 Visual perspective error proportions (bars = standard deviation) by condition and trial

There was no difference in either response time or error rate between the participants taking their own perspective (self condition) or taking the avatar’s perspective (other condition), indicating that in general there was no difficulty in taking one perspective over another. Other perspective trials show higher response times and error rates than self perspective trials, possibly due to greater interference of the self perspective in the inconsistent trials only.

This suggests that the L1 VPT was showing similar patterns to the original study of Samson et al. (2010).

The critical dependent variables for the task were calculated as per Bukowski and Samson (2017), resulting in a conflict index (inconsistent–consistent perspectives) and a focus Index (self perspective–other perspective), using inverse efficiency scores (IES; response time / 1 − error rate).^[[5]](#footnote-5)^ A higher value in the conflict index indicated greater difficulty in handling conflicting perspectives, while positive values in the focus index indicated better performance in taking the other person’s perspective than the self perspective (more altercentric rather than egocentric). Results showed that the mean conflict index for participants was .21 (*SD* = .13), with an excellent reliability of .85, and the mean focus index was −.004 (*SD* = .13), with again an excellent reliability of .88. The variances were adjusted to within a 10:1 ratio of the other task measures (Kline, 2005).

**Director task**

For the response time analyses, the data were trimmed by condition for each participant individually, looking at correct responses only (12.5% were incorrect responses to the competitor item, 1.4% were responses to another object). Response time values that were more than three standard deviations away from the condition mean for a participant were replaced by the cutoff value (of three standard deviations from the condition mean). This was done for 0.48% of trials. The data were analyzed using a 2 × 2 repeated-measures ANOVA, with trial type (control vs. experimental) and condition (ambiguous vs. relational) as variables. Response time was looked at first, with correct responses only used. There was no effect of trial type, *F*(1, 141) = .12, *p* = .73, η_p_^2^ = .00. There was an effect of condition, *F*(1, 141) = 187.23, *p* < .01, η_p_^2^ = .59, with the response time to ambiguous trials being significantly faster than to relational trials (see Fig. 13). There was also a marginal significant interaction between trial type and condition, *F*(1, 141) = 3.71, *p* = .06, η_p_^2^ = .02. These findings, compared against the error findings described below, show no indication of a speed–accuracy trade-off.

Fig. 13 Mean response times (and standard error) in the director task by condition and trial type

The error rate was then examined with a series of *t* tests, as no errors made in the control conditions (error data were not trimmed). A one-sample *t* test (with a theoretical value of zero) showed that significantly more errors than zero were made in the experimental condition, *t*(141) = 12.89, *p* < .01, with a mean difference of 3.94 (*SD* = 3.59). A paired-samples *t* test showed there was also an effect of condition in the experimental trials, t(141) = −8.88, *p* < .01, with significantly more errors in the relational condition than in the ambiguous condition.

For the ambiguous trials there were significantly more errors than zero, *t*(141) = 11.36, *p* < .01, with a mean difference of 1.17 (*SD* = 1.21). There were significantly more errors than zero in the relational trials, *t*(141) = 12.12, *p* < .01, with a mean difference of 2.77 (*SD* = 2.68). These differences can be seen in Fig. 14.

Fig. 14 Mean number of errors (and standard error) in Director task by condition and trial type

The control condition was expected to have no errors and a faster response time due to there being no competing item for the item description given by the instructor. The relational trials were slower and resulted in more errors than the ambiguous trials. This may be due to there being more items that the participant is cued to in the relational trials (for example, in the experimental grid where the critical instruction is “move the small ball . . . ,” and there are three different sized balls present) compared with in the ambiguous trials (where in an experimental grid, where the critical instruction is “move the mouse . . .,” and there is a computer mouse and a mouse, so there are only two potential items). The additional item that the participant must consider in relational trials may explain the increased response times, and perhaps the increased error rate.

The critical dependent variables for the director task were the error rates of the relational and ambiguous trials. These were expected to measure the participants’ failure to use their theory of mind. Both the distributions of the dependent variables were skewed, and they were both square-root transformed (ambiguous with a constant of one to remove negative values). This resulted in adequately normal distributions for both dependent variables. The reliability of the ambiguous measure was borderline satisfactory (.59), though the reliability of the relational measure was excellent (.83).

**Appendix A4**

**Executive task discussion**

An initial correlation matrix including the Simon task is shown in Table 5. There were no correlations between the Simon task and any other task, so this was dropped from the final analyses.

Table 5. Correlation matrix between variables

|  | Simon task | Shape matching | Go/no-go | Go/no-go (picture) | Stop signal | Director task (ambiguous) | Director task (relational) | Visual perspective (conflict) |
| --- | --- | --- | --- | --- | --- | --- | --- | --- |
| Shape matching | .07 |  |  |  |  |  |  |  |
| Go/no-go | .11 | −.04 | – |  |  |  |  |  |
| Go/no-go (picture) | .03 | .00 | .23** | – |  |  |  |  |
| Stop signal | .14 | .15 | .18* | .08 | – |  |  |  |
| Director (ambiguous) | −.09 | .09 | .00 | .34** | .02 | – |  |  |
| Director (relational) | .04 | .29** | .01 | .25** | .05 | .65** | – |  |
| Visual perspective (Conflict) | .12 | .06 | .14 | .00 | .24** | −.03 | −.04 | – |
| Visual perspective (Focus) | .07 | −.02 | .08 | −.04 | .02 | .02 | −.03 | −.18* |

Final results from the path analyses showed a correlation between the go/no-go (picture) and go/no-go tasks, and between the go/no-go (picture) and the SST. There were no correlations with the shape-matching task. While we predicted that the executive tasks would all intercorrelate as they all require response-distracter inhibition (as per Friedman & Miyake, 2004), the specific nature of the inhibition required by these varying tasks may explain this pattern of correlations. The go/no-go tasks both involve selecting relevant *responses—*that is to respond (go) or not (no-go) to the stimuli. Likewise the SST involves selecting the appropriate response to a given stimuli—that is, whether to respond (go) or inhibit their response (stop) depending on whether there is a stop signal. On the other hand, the shape-matching task involves selection of relevant and ignoring of irrelevant *information*, whereby the participant needs to ignore the distracter shape and select their response based on the relevant target shape. This may explain why responses on shape matching were not related with the other executive measures.

The nature of the ToM tasks may also explain the relationships found with the different executive tasks. As noted, L1 VPT performance, particularly on the conflict index, may be related to the effect of self–other consistency on selection of a response (Apperly, 2010; McCleery et al., 2011; Qureshi et al., 2010), rather than on selection of information or any integration. As such, it is perhaps unsurprising that conflict index of the L1 VPT is only related to the SST. However, the director task requires integration of perspective and instruction, which may involve selection of relevant information and also response selection. This perspective would explain the relationship between to the go/no-Go (picture) and shape-matching task. The latter may be related to relational trials of the director task due to there being potentially three referent objects (rather than two as in ambiguous trials).

**References**

Apperly, I. A. (2010). *Mindreaders: The cognitive basis of “theory of mind.”* London, UK: Psychology Press.

Apperly, I. A., Carroll, D. J., Samson, D., Qureshi, A., Humphreys, G. W. & Moffatt, G. (2010). Why are there limits on theory of mind use? Evidence from adults’ ability to follow instructions from an ignorant speaker. *Quarterly Journal of Experimental Psychology*, *63*(6), 1201–1217.

Barr, D. (2008). Pragmatic expectations and linguistic evidence: Listeners anticipate but do not integrate common ground. *Cognition,* 109(1), 18–40.

DeSchepper, B., & Treisman, A. (1996). Visual memory for novel shapes: Implicit coding without attention. *Journal of Experimental Psychology: Learning, Memory & Cognition, 22*(1), 27–47.

Endo, N., Saiki, J., & Saito, H. (2001). Determinants of occurrence of negative priming for novel shapes with matching paradigm. *Japanese Journal of Psychology, 72*(3), 204–212.

Friedman, N. P., & Miyake, A. (2004). The relations among inhibition and interference control functions: A latent-variable analysis. *Journal of Experimental Psychology: General, 133*(1), 101–135.

Kline, R. B. (2005). *Principles and practice of structural equation modelling* (2nd ed.). New York, NY: Guilford Press.

McCleery, J. P., Surtees, A. D. R., Graham, K. A., Richards, J. E. & Apperly, I. A. (2011). The neural and cognitive time course of theory of mind. *The Journal of Neuroscience,* *31*(36), 12849–12854.

Oberauer, K. (2005). Binding and inhibition in working memory: Individual and age differences in short-term recognition. *Journal of Experimental Psychology: General*, *134*(3), 368–387.

Peterson, B. S., Kane, M. J., Alexander, G. M., Lacadie, C., Skudlarski, P., Leung, H.-C., . . . Gore, J. C. (2002). An event-related functional MRI study comparing interference effects in the Simon and Stroop tasks. *Cognitive Brain Research, 13,* 427–440.

Qureshi, A. W., Apperly, I. A., & Samson, D. (2010). Executive function is necessary for perspective selection, not Level-1 visual perspective calculation: Evidence from a dual-task study of adults. *Cognition*, *117*, 230–236.

Salthouse, T. A., Siedlecki, K. L., & Krueger, L. E. (2006). An individual differences analysis of memory control. *Journal of Memory and Language, 55*(1), 102–125.

Schmitt, B. M., Münte, T. F., & Kutas, M. (2000). Electrophysiological estimates of the time course of semantic and phonological encoding during implicit picture naming. *Psychophysiology, 37,* 473–484.

Tabachnick, B. G., & Fidell, L. S. (2001). *Using multivariate statistics* (4th ed.). Boston, MA: Allyn & Bacon.

Tolan, G. A., & Tehan, G. (1999). Determinants of short-term forgetting: Decay, Retroactive interference, or proactive interference. *International Journal of Psychology, 34*(5/6), 285–292.

van Turennout, M., Hagoort, P., & Brown, C. M. (1997). Electrophysiological evidence on the time course of semantic and phonological processes in speech production*. Journal of Experimental Psychology: Learning, Memory & Cognition*, *23*(4), 787–806.

1. Participants were presented with eight serially presented random numbers of two digits (one per 1,000 ms), and had to judge aloud if they were above or below 50. [↑](#footnote-ref-1)
2. The participants were shown (serially) all the images of birds and mammals, together with their names, at the start of the experiment (each image remained on-screen until the participant pressed a key). They were then shown the images again, in a different order, for 3,000 +ms, in which time they were instructed to name each image. Pilot work had already shown people were familiar with the mammals and birds used. [↑](#footnote-ref-2)
3. For the L1 VPT we followed the procedure of Samson et al. (2010) and used 2.5 SD rather than 3SD). [↑](#footnote-ref-3)
4. These participants failed to complete the cued-recall task due to time constraints (arrived late to session). [↑](#footnote-ref-4)
5. Use of IES is not recommended if speed–accuracy trade-offs are shown (indicated by no positive correlation between response times and error rates) and if the average error rate is above .10 (cf. Bukowski & Samson, 2017). The correlation between response time and error rate was positive, *r*(142) = .20, *p* < .01, and the mean error rate across conditions was 0.06 (*SD* = 0.07), meaning both recommendations for using IES were met. [↑](#footnote-ref-5)
