# Supplementary material for: Does interference between self and other perspectives in theory of mind tasks reflect a common underlying process? Evidence from individual differences in theory of mind and inhibitory control
Source: Psychon Bull Rev. 2019 Aug 19;27(1):178–90. doi: 10.3758/s13423-019-01656-z (PMC7000534; doi:10.3758/s13423-019-01656-z)
Supplement: Supplementary file 2 — (DOCX 221 kb) [file 13423_2019_1656_MOESM2_ESM.docx]

**Appendix B (models prior to final)**

**Initial model**

The initial model shown in Fig. 15 showed no relationship between the go/no-go or stop-signal tasks and either of the director task variables. The shape-matching task only predicted performance on the relational variable of the director task. The go/no-go (picture) task predicted performance on both variables of the director task. The stop-signal task predicted performance on the L1 VPT conflict index. All parameters (standardized and unstandardized path coefficients, covariances and correlations, variances and squared multiple correlations) are shown in Table 6.


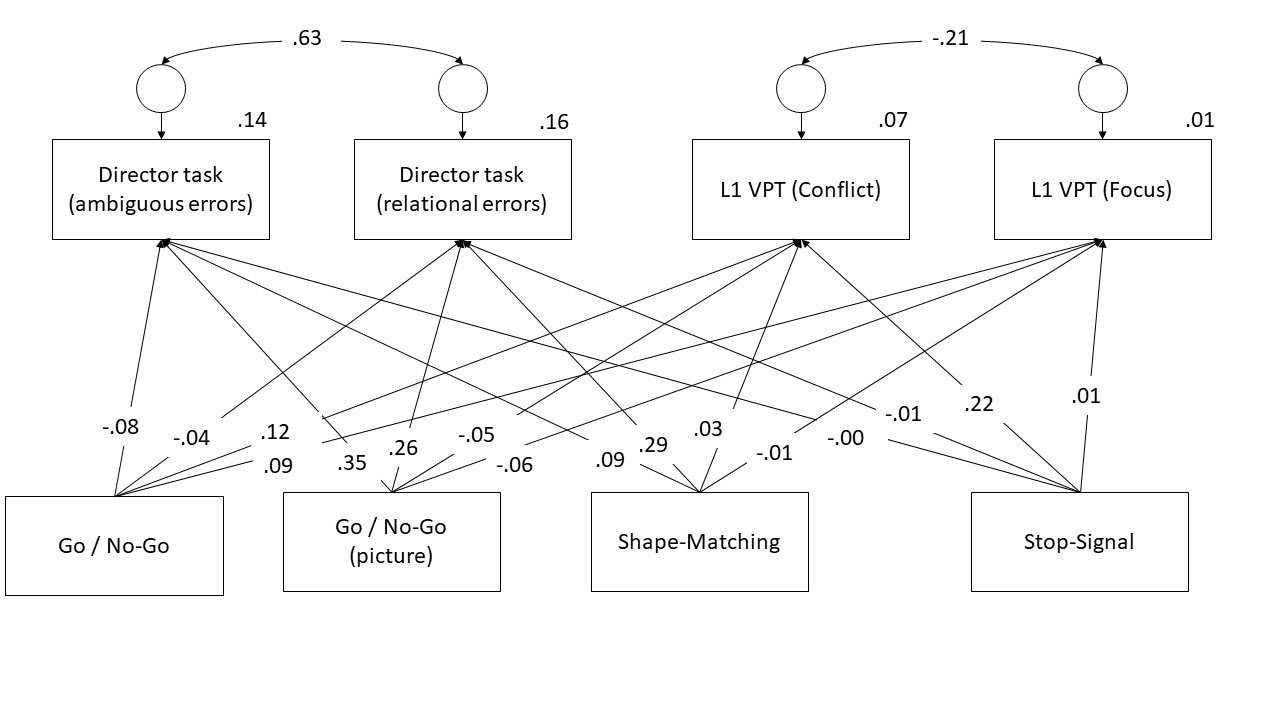


Fig. 15 Initial model (standardized coefficients)

Table 6 Parameter estimates for initial model

| **Path** | **Unstandardized estimate (*SE*)** | **Standardized estimate** |
| --- | --- | --- |
| Shape-matching → Director (relational) | .16 (.04) | .29*** |
| Go/no-go → Director (relational) | −.05 (.10) | −.04 |
| Go/no-go (picture) → Director (relational) | .23 (.07) | .26*** |
| SST → Director (relational) | −.00 (.03) | −.01 |
| Shape-matching →Director (ambiguous) | .04 (.04) | .09 |
| Go/no-go → Director (ambiguous) | −.09 (.09) | −.08 |
| Go/no-go (picture) → Director (ambiguous) | .28 (.06) | .35*** |
| SST → Director (ambiguous) | −.00 (.03) | −.00 |
| Shape-matching →L1 VPT (focus) | −.01 (.04) | −.02 |
| Go/no-go → L1 VPT (Focus) | .09 (.08) | .09 |
| Go/no-go (picture) → L1 VPT (focus) | −.04 (.06) | −.06 |
| SST → L1 VPT (focus) | .00 (.03) | .01 |
| Shape-matching → L1 VPT (conflict) | .02 (.04) | .03 |
| Go/no-go → L1 VPT (conflict) | .12 (.09) | .12 |
| Go/no-go (picture) → L1 VPT (conflict) | −.03 (.06) | −.05 |
| SST → L1 VPT (conflict) | .07 (.03) | .22** |
|  |  |  |
| **Covariances** | **Unstandardized estimate (*SE*)** | **Standardized estimate** |
| Director (relational) ↔ Director (ambiguous) | 3.42 (.54) | .63*** |
| L1 VPT (conflict) ↔L1 VPT (focus) | −.93 (.39) | −.21* |
|  |  |  |
| **Variances** | **Estimate (*SE*)** |  |
| Shape-matching | 23.60 (2.81)*** |  |
| Go/no-go | 4.48 (.53)*** |  |
| Go/no-go (picture) | 9.22 (1.10)*** |  |
| SST | 45.20 (5.38)*** |  |
| e1 (Director (relational)) | 5.96 (.71)*** |  |
| e2 (Director (ambiguous)) | 5.02 (.60) *** |  |
| e3 (L1 VPT (conflict)) | 4.47 (.53)*** |  |
| e4 (L1 VPT (focus)) | 4.66 (.56)*** |  |
|  |  |  |
| **Squared Multiple Correlations** |  |  |
| Director (relational) | .16 |  |
| Director (ambiguous) | .14 |  |
| L1 VPT (conflict) | .07 |  |
| L1 VPT (focus) | .01 |  |

**p* < .05. ***p* < .01. ****p* < .001

Table 6 shows significant correlations between the pairs of variables for each ToM task, and that the error terms and variances were all significant. Approximately 16% of the relational variable and 14% of the ambiguous variable were accounted for by this initial model. The model (Table 5) however, was not a good fit to the actual data.

**Model 2**

Nonsignificant paths were removed, resulting in the model shown in Fig. 16.


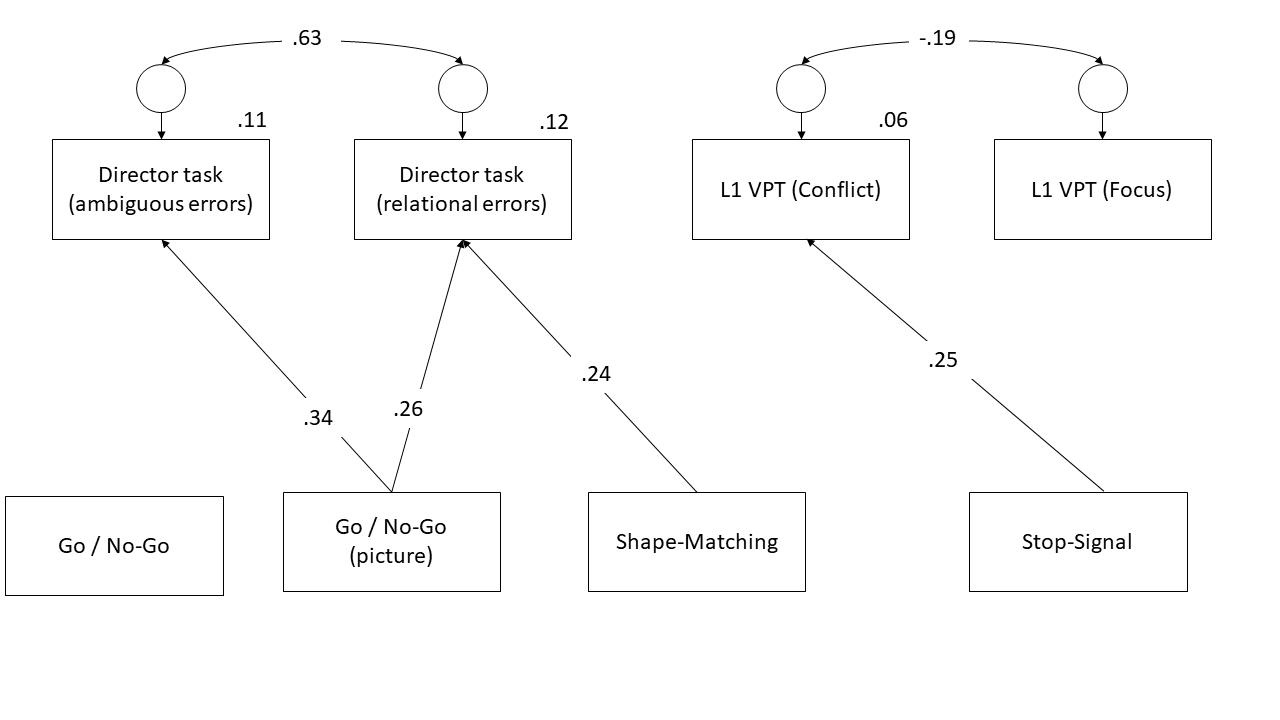


Fig. 16 Model 2 (standardized coefficients)

Table 7 shows the model parameters in full. Removing the non-significant parameters did not affect the individual parameters or the model fit (Table 5), with the AIC value reducing from the initial model.

Table 7 Parameter estimates for Model 2

| **Path** | **Unstandardized estimate (*SE*)** | **Standardized estimate** |
| --- | --- | --- |
| Shape-matching →Director (relational) | .13 (.03) | .24*** |
| Go/no-go → Director (relational) |  |  |
| Go/no-go (picture) → Director (relational) | .22 (.07) | .26*** |
| SST → Director (relational) |  |  |
| Shape-matching →Director (ambiguous) |  |  |
| Go/no-go → Director (ambiguous) |  |  |
| Go/no-go (picture) → Director (ambiguous) | .26 (.06) | .34*** |
| SST → Director (ambiguous) |  |  |
| Shape-matching →L1 VPT (focus) |  |  |
| Go/no-go → L1 VPT (focus) |  |  |
| Go/no-go (picture) → L1 VPT (focus) |  |  |
| SST → L1 VPT (focus) |  |  |
| Shape-matching → L1 VPT (conflict) |  |  |
| Go/no-go → L1 VPT (conflict) |  |  |
| Go/no-go (picture) → L1 VPT (conflict) |  |  |
| SST → L1 VPT (conflict) | .08 (.03) | .25** |
|  |  |  |
| **Covariances** | **Unstandardized estimate (*SE*)** | **Standardized estimate** |
| Director (relational) ↔ Director (ambiguous) | 3.54 (.56) | .63*** |
| L1 VPT (conflict) ↔L1 VPT (focus) | −.89 (.40) | −.19* |
|  |  |  |
| **Variances** | **Estimate (*SE*)** |  |
| Shape-matching | 23.60 (2.81)*** |  |
| Go/No-Go | 4.48 (.53)*** |  |
| Go/no-go (picture) | 9.22 (1.10)*** |  |
| SST | 45.20 (5.38)*** |  |
| e1 (Director (relational)) | 5.99 (.71)*** |  |
| e2 (Director (ambiguous)) | 5.10 (.61)*** |  |
| e3 (L1 VPT (conflict)) | 4.51 (.54)*** |  |
| e4 (L1 VPT (focus)) | 4.73 (.56)*** |  |
|  |  |  |
| **Squared multiple correlations** |  |  |
| Director (relational) | .12 |  |
| Director (ambiguous) | .11 |  |
| L1 VPT (conflict) | .06 |  |
| L1 VPT (focus) | .00 |  |

**p* < .05. ***p* < .01. ****p* < .001
