# Supplementary material for: Does interference between self and other perspectives in theory of mind tasks reflect a common underlying process? Evidence from individual differences in theory of mind and inhibitory control
Source: Psychon Bull Rev. 2019 Aug 19;27(1):178–90. doi: 10.3758/s13423-019-01656-z (PMC7000534; doi:10.3758/s13423-019-01656-z)
Supplement: Supplementary file 3 — (DOCX 333 kb) [file 13423_2019_1656_MOESM3_ESM.docx]

**Appendix C (all models)**

The following analyses used interference measures for self (altercentric interference from the avatar perspective) and other (egocentric interference from the self perspective) for the L1 VPT task. These were calculated based on reaction times for correct trials only and were “inconsistent perspective condition–consistent condition” for self and other trials separately.

After variance adjustment and transformations, the final descriptive statistics for the dependent and independent variables were as shown in Table 8.

Table 8 Final task descriptives

| **Measure** | ***M* (*SD*)** | **Variance** | **Reliability^[[1]](#footnote-1)^** |
| --- | --- | --- | --- |
| Shape-matching (ms) (square-root transformation) | 21.56 (4.88) | 23.77 | .92 |
| Go/no-go (FAR) | 4.28 (2.12) | 4.51 | .93 |
| Go/no-go (picture) (FAR; Bird/Mammal)^[[2]](#footnote-2)^ | 6.17 (3.05) | 9.28 | .74 |
| Stop signal (SSRT) (log 10 transformation) | 16.33 (6.74) | 45.52 | .99 |
| Director task (errors) (log 10 transformation)  Ambiguous  Relational | 2.34 (2.41)  2.75 (2.66) | 5.79  7.07 | .59  .83 |
| Visual perspective measures (ms)  Egocentric interference  Altercentric interference | 4.88 (5.62)  8.52 (6.33) | 31.63  40.08 | .63  .43 |

An initial correlation matrix between the variables is shown in Table 9. For the inhibitory control tasks, positive correlations were shown between the shape-matching and stop-signal tasks, the go/no-go and go/no-go (picture) tasks, and the go/no-go and stop-signal tasks. A positive correlation was shown between the director task variables, and a negative one between the L1 VPT variables. The shape-matching task had positive correlations with both the director (relational) variable, while the go/no-go (picture) task had positive correlations with both measures of the director task. The stop-signal task also had a positive correlation with the L1 VPT (conflict) variable.

Table 9 Correlation matrix between variables

|  | Shape matching | Go/no-go | Go/no-go (picture) | Stop signal | Director task (ambiguous) | Director task (relational) | Visual perspective (conflict) |
| --- | --- | --- | --- | --- | --- | --- | --- |
| Go/no-go | −.04 | – |  |  |  |  |  |
| Go/no-go (picture) | .00 | .23** | – |  |  |  |  |
| Stop signal | .15 | .18* | .08 | – |  |  |  |
| Director (ambiguous) | .09 | .00 | .34** | .02 | – |  |  |
| Director (relational) | .29** | .01 | .25** | .05 | .65** | – |  |
| Visual perspective (egocentric) | .19* | .10 | .04 | .24** | .05 | .08 | – |
| Visual perspective Altercentric | .16 | .03 | .01 | .14 | −.12 | −.07 | .24** |

* *p* < .05, ** *p* < .01

**Models**

Analyses were carried out using AMOS 25.

***Initial model***

The initial model shown in Fig. 17 showed no relationship between the go/no-go or stop-signal tasks and either of the director task variables. The shape-matching task predicted performance on the relational variable of the director task. The go/no-go (picture) task predicted performance on both variables of the director task. The stop-signal task and shape-matching task predicted performance egocentric interference. All parameters (standardized and unstandardized path coefficients, covariances and correlations, variances and squared multiple correlations) are shown in Table 10.


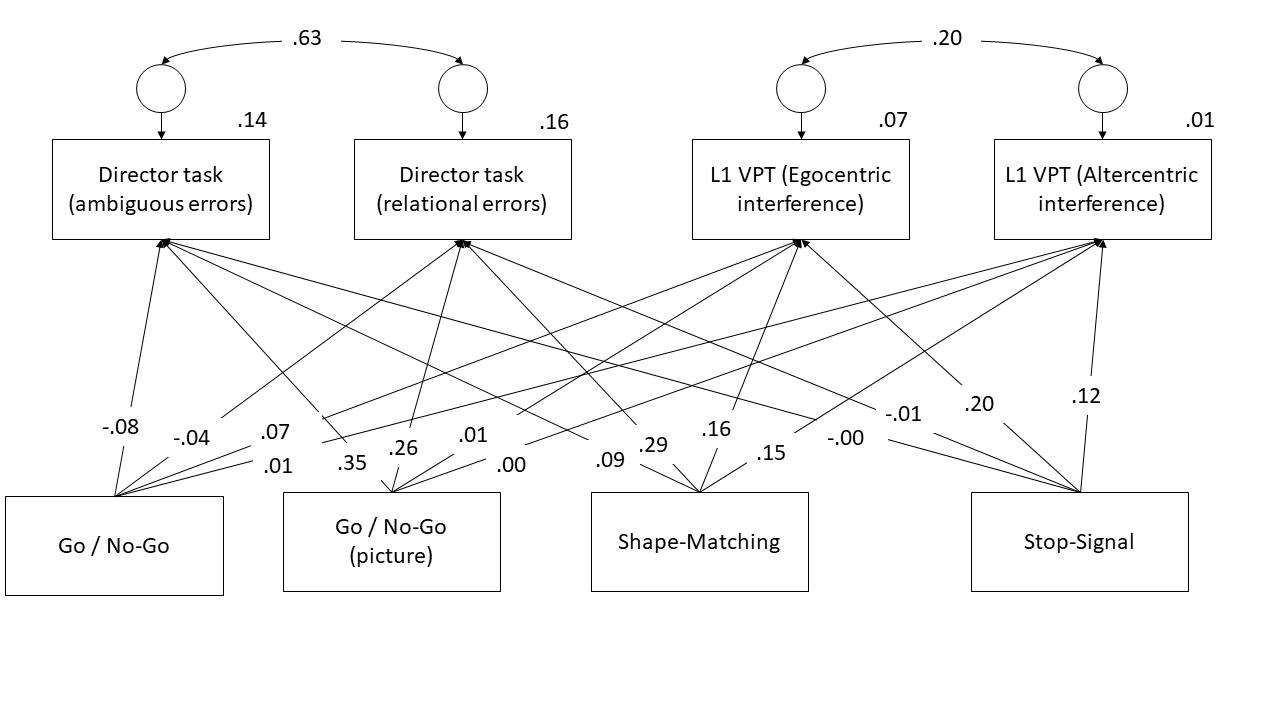


Fig. 17 Initial model (standardized coefficients)

Table 10. Parameter estimates for initial model

| **Path** | **Unstandardized estimate (*SE*)** | **Standardized estimate** |
| --- | --- | --- |
| Shape-matching → Director (relational) | .16 (.04) | .29*** |
| Go/no-go → Director (relational) | −.05 (.10) | −.04 |
| Go/no-go (picture) → Director (relational) | .23 (.07) | .26*** |
| SST → Director (relational) | −.00 (.03) | −.01 |
| Shape-matching →Director (ambiguous) | .04 (.04) | .09 |
| Go/no-go → Director (ambiguous) | −.09 (.09) | −.08 |
| Go/no-go (picture) → Director (ambiguous) | .28 (.06) | .35*** |
| SST → Director (ambiguous) | −.00 (.03) | −.00 |
| Shape-matching →L1 VPT (altercentric interference) | .17 (.10) | .15 |
| Go/no-go → L1 VPT (altercentric interference) | .04 (.22) | .01 |
| Go/no-go (picture) → L1 VPT (altercentric interference) | .00 (.15) | .00 |
| SST → L1 VPT (altercentric interference) | .10 (.07) | .12 |
| Shape-matching → L1 VPT (egocentric interference) | .21 (.11) | .16* |
| Go/no-go → L1 VPT (egocentric interference) | .20 (.24) | .07 |
| Go/no-go (picture) → L1 VPT (egocentric interference) | .02 (.17) | .01 |
| SST → L1 VPT (egocentric interference) | .19 (.08) | .20* |
|  |  |  |
| **Covariances** | **Unstandardized estimate (*SE*)** | **Standardized estimate** |
| Director (relational) ↔ Director (ambiguous) | 3.42 (.54) | .63*** |
| L1 VPT (egocentric interference) ↔ L1 VPT (altercentric interference) | 6.57 (.2.84) | .20* |
|  |  |  |
| **Variances** | **Estimate (*SE*)** |  |
| Shape-matching | 23.60 (2.81)*** |  |
| Go/no-go | 4.48 (.53)*** |  |
| Go/no-go (picture) | 9.22 (1.10)*** |  |
| SST | 45.20 (5.38)*** |  |
| e1 (Director (relational)) | 5.96 (.71)*** |  |
| e2 (Director (ambiguous)) | 5.02 (.60) *** |  |
| e3 (L1 VPT (egocentric interference)) | 30.11 (3.59)*** |  |
| e4 (L1 VPT (altercentric interference)) | 36.43 (4.34)*** |  |
|  |  |  |
| **Squared multiple correlations** |  |  |
| Director (relational) | .16 |  |
| Director (ambiguous) | .14 |  |
| L1 VPT (Egocentric interference) | .07 |  |
| L1 VPT (altercentric interference) | .04 |  |

**p* < .05. ***p* < .01. ****p* < .001

Table 10 shows significant correlations between the pairs of variables for each ToM task, and that the error terms and variances were all significant. Approximately 16% of the relational variable and 14% of the ambiguous variable were accounted for by this initial model. The model (see Table 4) however, was not a good fit to the actual data.

***Model 2***

Nonsignificant paths were removed, resulting in the model shown in Fig. 18. Doing this did not significantly affect the model fit, *χ*^2^(11) = 8.85, *p* = .64.


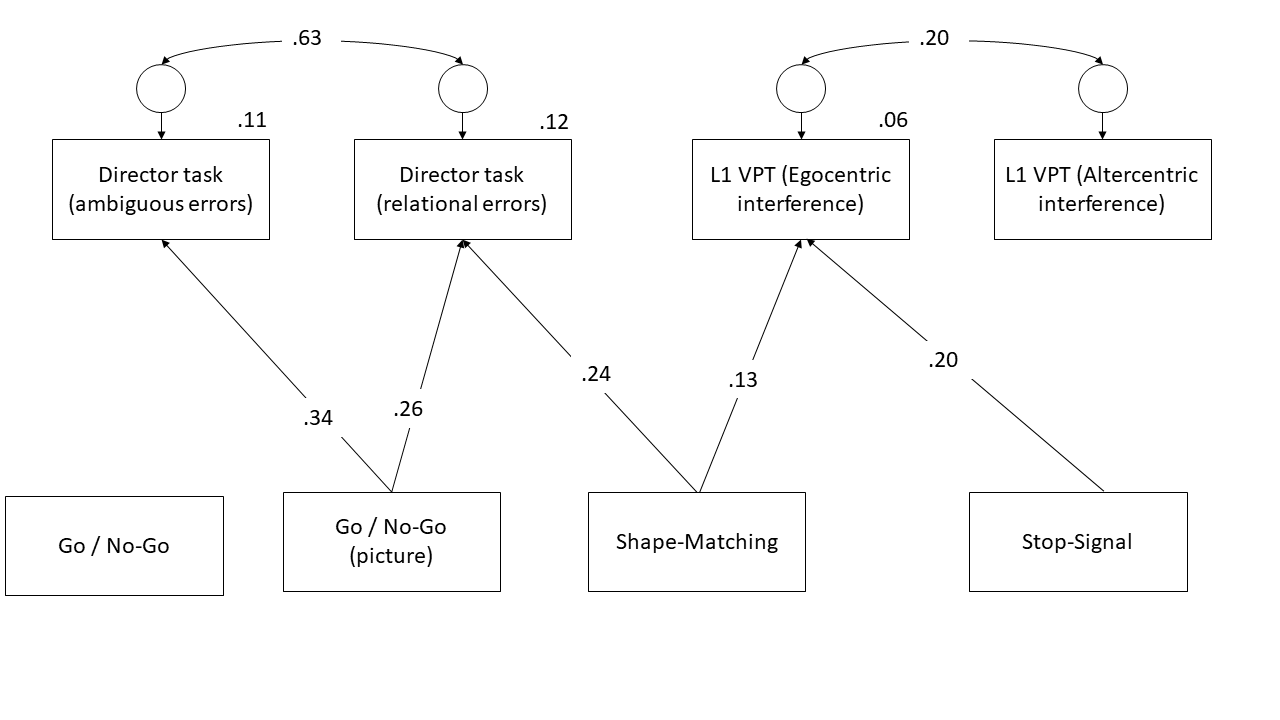


Fig. 18 Model 2 (standardized coefficients)

Table 11 shows the model parameters in full. Removing the non-significant parameters did not affect the individual parameters or the model fit (see Table 5), with the AIC value reducing from the initial model.

Table 11 Parameter estimates for Model 2

| **Path** | **Unstandardized estimate (*SE*)** | **Standardized estimate** |
| --- | --- | --- |
| Shape-matching → Director (relational) | .13 (.03) | .24*** |
| Go/no-go → Director (relational) |  |  |
| Go/no-go (picture) → Director (relational) | .22 (.07) | .26*** |
| SST → Director (relational) |  |  |
| Shape-matching → Director (ambiguous) |  |  |
| Go/no-go → Director (ambiguous) |  |  |
| Go/no-go (picture) → Director (ambiguous) | .26 (.06) | .34*** |
| SST → Director (ambiguous) |  |  |
| Shape-matching → L1 VPT (altercentric interference) |  |  |
| Go/no-go → L1 VPT (altercentric interference) |  |  |
| Go/no-go (picture) → L1 VPT (altercentric interference) |  |  |
| SST → L1 VPT (Altercentric interference) |  |  |
| Shape-matching → L1 VPT (egocentric interference) | .16 (.10) | .13 |
| Go/no-go → L1 VPT (Egocentric interference) |  |  |
| Go/no-go (picture) → L1 VPT (egocentric interference) |  |  |
| SST → L1 VPT (egocentric interference) | .18 (.07) | .19* |
|  |  |  |
| **Covariances** | **Unstandardized estimate (*SE*)** | **Standardized estimate** |
| Director (relational) ↔ Director (ambiguous) | 3.47 (.55) | .63*** |
| L1 VPT (Egocentric interference) ↔ L1 VPT (Altercentric interference) | 6.89 (2.92) | .20* |
|  |  |  |
| **Variances** | **Estimate (*SE*)** |  |
| Shape-matching | 23.60 (2.81)*** |  |
| Go/no-go | 4.48 (.53)*** |  |
| Go/no-go (picture) | 9.22 (1.10)*** |  |
| SST | 45.20 (5.38)*** |  |
| e1 (Director (relational)) | 5.99 (.71)*** |  |
| e2 (Director (ambiguous)) | 5.10 (.61)*** |  |
| e3 (L1 VPT (egocentric interference)) | 31.40 (3.74)*** |  |
| e4 (L1 VPT (altercentric interference)) | 36.68 (4.37)*** |  |
|  |  |  |
| **Squared Multiple Correlations** |  |  |
| Director (relational) | .12 |  |
| Director (ambiguous) | .11 |  |
| L1 VPT (egocentric interference) | .05 |  |
| L1 VPT (altercentric interference) | .00 |  |

**p* < .05. ***p* < .01. *** *p* < .001

***Model 3 (final)***

The nonsignificant path between the shape-matching task and egocentric interference was removed, and correlations added between the go/no-go (picture) and go/no-go tasks and also between the go/no-go and stop-signal tasks (as per modification indices), resulting in the final model in Fig. 19. The model fit (as per AIC values) was improved.


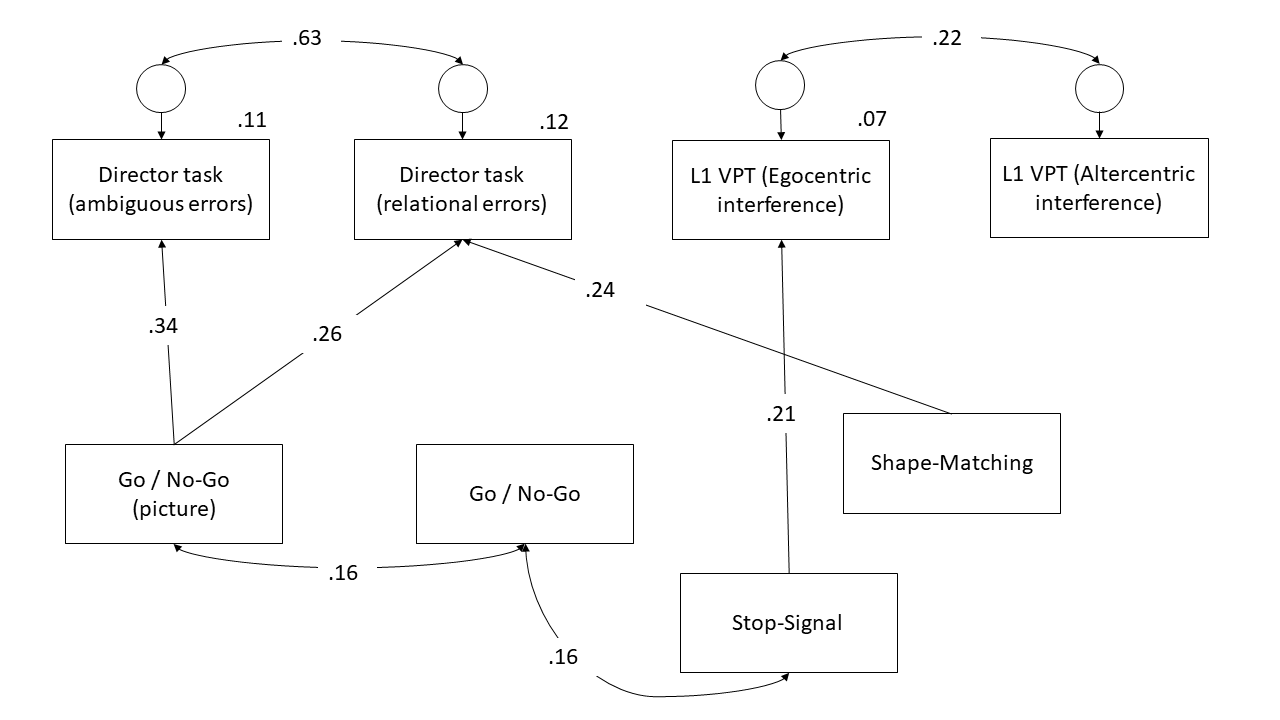


Fig. 19 Final model (standardized coefficients)

Table 12 shows the model parameters in full.

Table 12 Parameter estimates for Model 3 (final)

| **Path** | **Unstandardized estimate (*SE*)** | **Standardized estimate** |
| --- | --- | --- |
| Shape-matching → Director (relational) | .13 (.03) | .24*** |
| Go/no-go → Director (relational) |  |  |
| Go/no-go (picture) → Director (relational) | .22 (.07) | .26*** |
| SST → Director (relational) |  |  |
| Shape-matching → Director (ambiguous) |  |  |
| Go/no-go → Director (ambiguous) |  |  |
| Go/no-go (picture) → Director (ambiguous) | .26 (.06) | .34*** |
| SST → Director (ambiguous) |  |  |
| Shape-matching → L1 VPT (altercentric interference) |  |  |
| Go/no-go → L1 VPT (altercentric interference) |  |  |
| Go/no-go (picture) → L1 VPT (altercentric interference) |  |  |
| SST → L1 VPT (altercentric interference) |  |  |
| Shape-matching → L1 VPT (egocentric interference) |  |  |
| Go/no-go → L1 VPT (egocentric interference) |  |  |
| Go/no-go (picture) → L1 VPT (egocentric interference) |  |  |
| SST → L1 VPT (egocentric interference) | .20 (.08) | .21** |
|  |  |  |
| **Covariances** | **Unstandardized estimate (*SE*)** | **Standardized estimate** |
| Director (relational) ↔ Director (ambiguous) | 3.47 (.55) | .63*** |
| L1 VPT (egocentric interference) ↔L1 VPT (altercentric interference) | 7.52 (2.96) | .22* |
| Go/no-go ↔ SST | 2.29 (1.18) | .16* |
| Go/no-go ↔ Go/no-go (picture) | 1.38 (.55) | .22* |
|  |  |  |
| **Variances** | **Estimate (*SE*)** |  |
| Shape-matching | 23.60 (2.81)*** |  |
| Go/no-go | 4.48 (.53)*** |  |
| Go/no-go (picture) | 9.22 (1.10)*** |  |
| SST | 45.20 (5.38)*** |  |
| e1 (Director (relational)) | 5.99 (.71)*** |  |
| e2 (Director (ambiguous)) | 5.10 (.61)*** |  |
| e3 (L1 VPT (egocentric interference)) | 31.40 (3.74)*** |  |
| e4 (L1 VPT (altercentric interference)) | 37.57 (4.47)*** |  |
|  |  |  |
| **Squared multiple correlations** |  |  |
| Director (relational) | .12 |  |
| Director (ambiguous) | .11 |  |
| L1 VPT (egocentric interference) | .04 |  |
| L1 VPT (altercentric interference) | .00 |  |

**p* < .05. ***p* < .01. ****p* < .001

Model fit values are shown in Table 13.

Table 13 Model fit parameters

|  | CMIN | *df* | *p* | NFI | CFI | AIC | RMSEA | Low | High | Bollen–Stine Bootstrap *p* |
| --- | --- | --- | --- | --- | --- | --- | --- | --- | --- | --- |
| Model 1 | 20.32 | 10 | <.05 | .87 | .92 | 72.32 | .09 | .03 | .14 | .06 |
| Model 2 | 29.17 | 21 | .11 | .81 | .94 | 59.17 | .05 | .00 | .10 | .18 |
| Model 3 | 20.16 | 20 | .45 | .87 | .99 | 52.16 | .01 | .00 | .07 | .52 |

1. Split-half reliabilities/ [↑](#footnote-ref-1)
2. As FAR for the bird and mammal trials were significantly correlated and followed the same pattern, they were collapsed to form a single FAR for the go/no-go (picture) task. [↑](#footnote-ref-2)
